# Supplementary material for: Neuromorphic overparameterisation and few-shot learning in multilayer physical neural networks
Source: Nat Commun. 2024 Aug 27;15:7377. doi: 10.1038/s41467-024-50633-1 (PMC11350220; doi:10.1038/s41467-024-50633-1)
Supplement: Supplementary file 1 — Supplementary Information [file 41467_2024_50633_MOESM1_ESM.pdf]

Supplementary Information

Supplementary note 1 - Ferromagnetic resonance spectroscopy and magnetic force microscopy images of the artificial spin systems

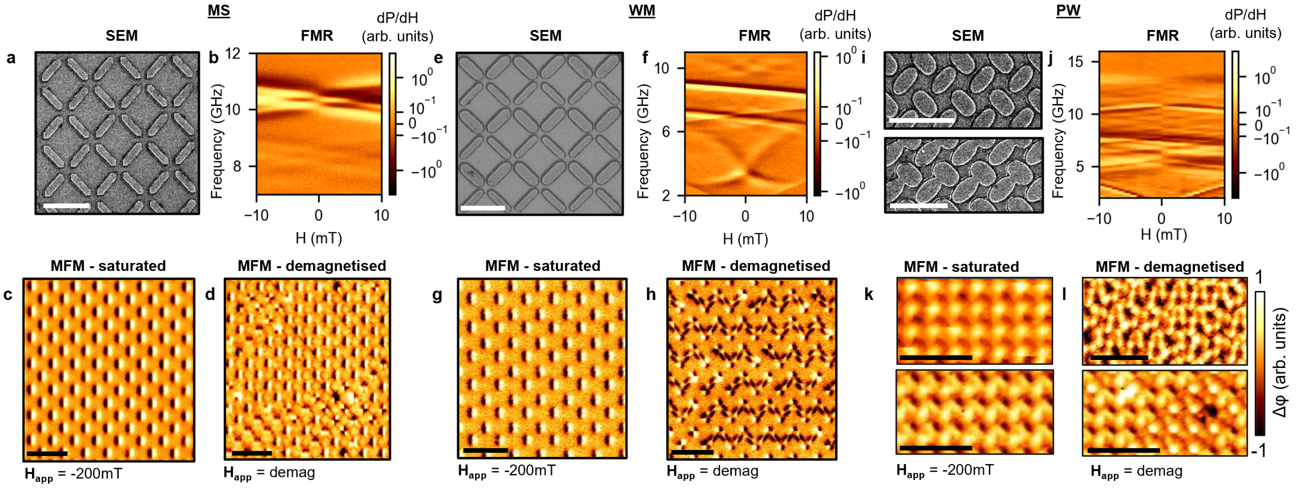

Supplementary Figure 1. Artificial spin system FMR response and bar states. MS is shown in a-d), WM in e-h), PW in i-l). a,e,i) Scanning electron micrographs (SEM) of each aarray. b,h,n) Ferromagnetic resonance (FMR) heatmaps measured after AC-demagnetisation. MS (b) shows linear macrospin modes. WM (f) shows linear macrospin modes (6-7 GHz wide-bar, 8-9 GHz thin bar) and nonlinear vortex modes (2-6 GHz). PW (j) exhibits rich linear and nonlinear modes. Magnetic force microscopy (MFM) images taken at remanence after field-saturation (c,g,k) and AC-demagnetisation (d,h,l). When saturated, all samples contain only macrospins. When demagnetised, WM and PW (h,l) show vortices.

The nanomagnetic arrays in this work are based on square and pinwheel artificial spin ice[1]. Supplementary Figure 1 shows scanning electron micrographs (SEM) (a,c,e) and ferromagnetic resonance (FMR) spectra after AC-demagnetisation (b,d,f). Three arrays were fabricated:

MS is a square artificial spin ice (Supplementary Figure 1 a,b). Bars are high aspect-ratio (530 nm × 120 nm) and only support macrospin states[2]. WM is a width-modified artificial spin-vortex ice with a subset of wider, lower-coercivity bars (Supplementary Figure 1 c,d). Bars are 600 nm × 200 nm (wide-bar)/125 nm (thin-bar). Wide bars host both macrospin and vortex states[2] whereas thin bars host just macrospins. PW is a pinwheel-lattice artificial spin-vortex ice (Supplementary Figure 1 e,f)[3,4] with higher density and inter-island coupling. A gradient of bar dimensions are patterned across the sample, ranging from fully-disconnected (e, top) to partially-connected islands (e, bottom) giving a complex range of spectral-responses (Supplementary Figure 1 f). Bar dimensions are constant across 100 × 100 μm^2 (length 450 nm, width 240 nm / 265 nm (lower / upper panel) in Supplementary Figure 1 e). Islands support macrospins and vortices.

MS FMR spectra comprise two dominant modes at 9.5 and 12 GHz with opposite linear gradients corresponding to macrospins aligned parallel (positive gradient) and anti-parallel (negative) to $\mathbf{H}_{app}$ (Supplementary Figure 1 b). In both the field-saturated (Supplementary Figure 1 c) and AC-demganetised state (Supplementary Figure 1 d), only macrospin states are observed due to the high aspect-ratio of these bars.

The WM FMR spectra comprises four dominant modes with rich responses: wide and thin bar linear macrospin modes (7 GHz and 9 GHz respectively), χ-shaped vortex mode (2-6 GHz) and 'whispering-gallery'-like high-frequency vortex mode[5] (10 GHz) (Supplementary Figure 1 f). Vortices are observed in the wide-bars when the sample is in an AC-demagnetised state.

PW displays a highly complex and non-linear FMR spectra (Supplementary Figure 1 j) due to the broad range of bar dimensions and state variations in this sample. Bars host both macrospins and vortices (Supplementary Figure 1 k,l)

Supplementary Figure 2 shows scanning electron micrographs of variation of dimensions across the MS (a,b) and PW (c-f) samples. In MS, two separate regions with widths of 124 nm and 137 nm are present. This manifests as slightly different coercive fields and resonant frequencies. In PW, a gradient of sample dimensions are fabricated across the surface of the chip. Bar subsets have slightly different widths to further enhance sample complexity. Widths range from 212 - 226 nm (thin) and 243 - 267 nm (wide) giving a broad distribution of resonant frequencies and coercive fields, enhancing output nonlinearity. Furthermore, some bars are connected (panel f) giving rise to complex magnetisation profiles and FMR spectral evolution.

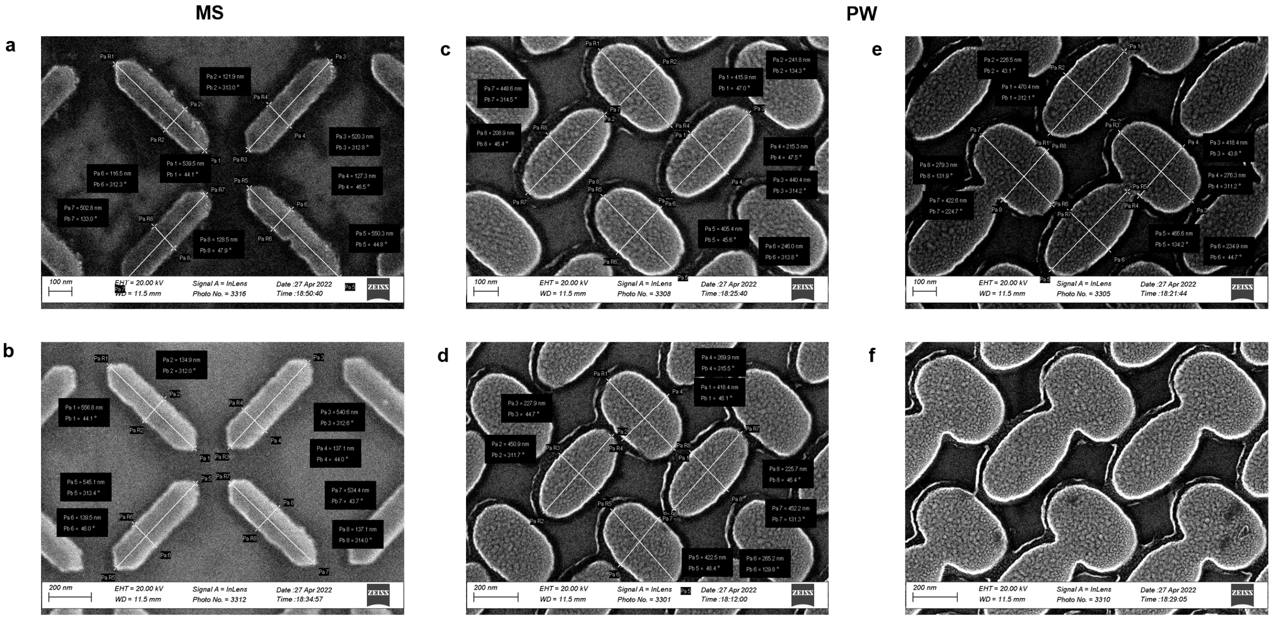

Supplementary Figure 2. Sample dimension variation. Variation of dimensions across the MS (a,b) and PW (c-f) samples.

Supplementary note 2 - k_{max} selection for metric calculation.

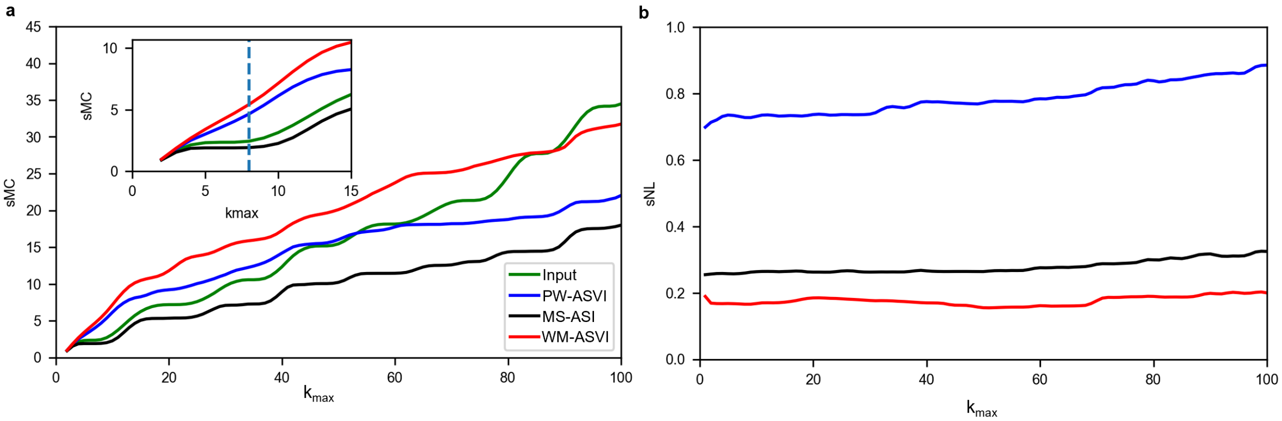

Supplementary Figure 3. Effects of k_{max} when calculating memory-capacity (MC) and nonlinearity (NL). a) memory-capacity (sMC) and b) nonlinearity (sNL) when varying how many previous inputs are used in the metric calculation (k_{max}). Memory-capacity profiles show characteristic humps and continual rising indicative of correlation between current and past input profiles from the periodic nature of the Mackey-Glass input equation.

Memory-capacity and nonlinearity are typically obtained by inputting a random input signal, with no correlation between consecutive inputs, to the reservoir. When calculating memory-capacity, this ensures that any observed memory effects arise from reservoir states alone. However, our measurement scheme precludes this method. Data is encoded in magnetic field amplitude and readout in-field resulting in field-dependent frequency shifts in the FMR response. For random inputs, the sharp jumps between consecutive inputs causes information to shift between outputs (e.g. shifts of 0.5 GHz spanning 25 output frequencies are observed over a 5 mT input range). Linear regression is unable to process this type of shifting leading to misleadingly low calculated memory-capacity.

As such, we use the smoothly varying Mackey-Glass equation as an input signal to calculate the metrics. This ensures that any field shifts between consecutive input are minimised. Our FMR peaks are broad with microstate information held

across multiple neighbouring outputs (e.g. 7 GHz and 7.02 GHz will be collinear). A disadvantage of this approach is that the Mackey-Glass equation is quasi-periodic. In addition to memory from the artificial spin reservoir states, this can lead to a memory arising from the similarity between current and previous inputs. As such, the value of k_{max} with which the metrics are calculated must be chosen carefully to minimise the effects of the periodic input.

Supplementary Figure 3 shows the memory-capacity and nonlinearity of the three artificial spin reservoir samples. Memory-capacity of the input signal with itself is also shown. All curves show characteristic humps and continuous rising in the memory-capacity profile due to the periodic nature of the MG input. Nonlinearity shows no variation with k_{max}. We chose $k_{max} = 8$ as this is at the end of the flat region on the input memory-capacity curve i.e. this is the maximum value before self-correlation from the next period starts.

Supplementary note 3 - Sine and NARMA transforms for each artificial spin reservoir

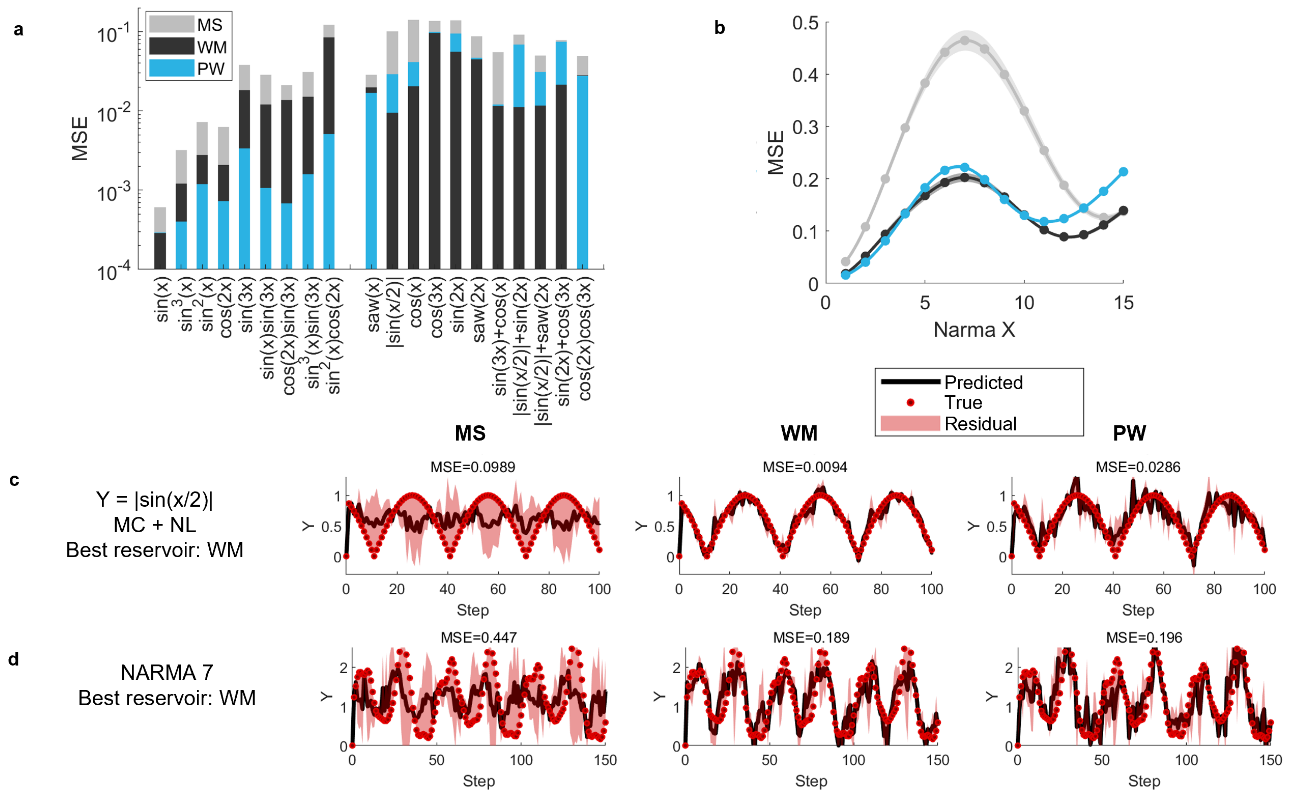

Supplementary Figure 4. Single reservoir sine and NARMA transforms. a) MSE when transforming a sine input to a variety of targets. Tasks are chosen to be symmetric (nonlinearity only) or asymmetric (memory-capacity + nonlinearity) w.r.t the input. Vortices enhance performance up to 31.6× for symmetric tasks. Higher memory-capacity enhances asymmetric transformations. b) MSE for NARMA-transformation on the Mackey-Glass input signal. X corresponds to how far back the NARMA model is evaluated on. Samples with high memory-capacity perform well. Example predictions for |sin(x/2)| and NARMA7 are shown in panels c) and d) respectively.

Supplementary Figure 4 a) shows the MSE for each reservoir when transforming a sinusoidal input to a variety of targets. Signal-transformations require varying memory-capacity and nonlinearity depending on the target waveform. Targets which are symmetric w.r.t. the input (eg. $\sin^2(x)$, sin(3x)sin(x)) require nonlinearity only, asymmetric waveforms w.r.t the input (e.g. saw(x), sin(x/2)) require both nonlinearity and memory-capacity as equivalent input values must be transformed to different output values across the wave-cycle. Highest nonlinearity PW dominates for symmetric transforms. Highest memory-capacity WM dominates for asymmetric transforms. Example plots for asymmetric |sin(x/2)| transform are shown in Supplementary Figure 4 c).

Supplementary Figure 4b) shows performance when performing a NARMA-transform[6] on the Mackey-Glass input, evaluated as $y(t) = Ay(t-1) + By(t-1)\sum_{n=1}^{X} y(t-n) + Cu(t-1)u(t-X) + D$ with A = 0.3, B = 0.01, C = 2, D = 0.1. X varies

between 1-15, defined as 'NARMAX'. This task adds additional nonlinear complexity vs. normal Mackey-Glass prediction, favouring both memory-capacity and nonlinearity with WM and PW performing similarly across all tasks. Example plots for the most-challenging NARMA7 are shown in Supplementary Figure 4 d).

Supplementary note 4 - Per-channel metric analysis for series connections

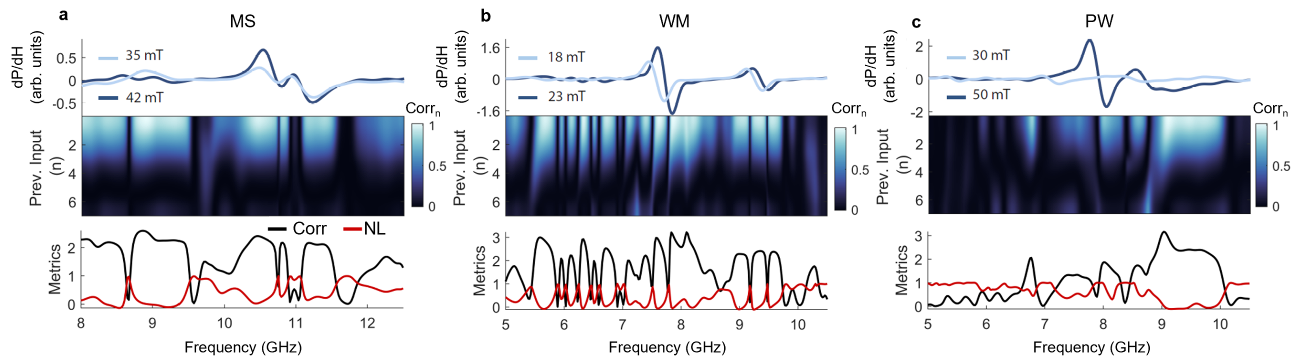

Supplementary Figure 5. Per-channel metric analysis. MS (a), WM (b) and PW (c) FMR amplitudes at maximum (dark blue) and minimum (light blue) input fields. Frequency-channel signal correlation ($Corr_n$) for previous time-steps (n) ranging from 0-7. Memory-capacity (black) and nonlinearity (red) across all output frequencies is shown. Corr is a sum of $Corr_n$ from n = 0 to 7.

We assess metrics on a per-output channel basis, highlighting that memory and nonlinearity are provided by distinct spectral channels. Figures 5 a-c) (top) show FMR spectra at max (dark blue) and min (light blue) input field-amplitude, correlation to previous time steps for 0-7 previous time-steps (n) where $Corr_n$ is the correlation to the nth previous input, total correlation and nonlinearity of each output frequency-channel (bottom). Here, the correlation calculation is identical to the memory-capacity calculation, except that only one channel is used rather than multiple channels. As such, we call it correlation to avoid confusion with other discussions of memory capacity. For MS, high $Corr_n$ is limited to $n < 3$. In contrast, WM and PW have some outputs which are correlated to 0-3 prior time-steps and others correlated to 4-7 steps (e.g. WM, 7.2 - 7.5 GHz). The presence of multiple correlation timescales is provided by vortex dynamics and is key to the strong prediction performance observed later. In PW, the main FMR mode has high nonlinearity due to complex disordered microstate dynamics. The gradient of physical structures in the array provide more non-degenerate nonlinear responses and hence the highest nonlinearity score.

Supplementary note 5 - Vortex induced memory amplification.

Supplementary Figures 6 a-c) show the memory-capacity of reservoir 1 (MC_{in}) vs memory-capacity of reservoir 2 (MC_{out})=when R2 is a) MS, b) WM and c) PW. Supplementary Figures 6 d-f) shows the relationship for MC_{in} vs MC_{out} when calculating memory-capacity for specific previous inputs. In all cases, a linear trend is observed. For MS, the gradient of MC_{in} vs MC_{out} stays approximately the same as memory-capacity is evaluated to further previous inputs. For WM and PW, strong MC_{out} is observed throughout. The gradient between input and output memory-capacity increases for further previous inputs. As such, the memory of the input signal is effectively amplified. The effect is strongest for WM. Small hints of long-term memory in the input signal translate to large contributions in the second reservoir. This provides additional information into why the ordering of reservoirs is crucial, as memory amplification at the end of the network is key for improved performance.

Supplementary note 6 - Interconnecting high-dimensional physical systems

No individual physical system excels across all tasks, and any single system tends to underperform for harder tasks. This is a well-known symptom of single reservoir systems in software and hardware[7–11]. In software, multiple reservoirs with distinct responses have been combined into networks to harness the benefits of different dynamical behaviours[7–11]. In such networks, each reservoir can be viewed as a complex node[12] with high-dimensionality and distinct memory and nonlinearity scores.

Here, we construct parallel and series (often termed hierarchical or deep) networks of physical reservoirs, combining a synergistic suite of distinct nanomagnetic arrays with substantially enhanced performance. The increased output dimensionality gained from multiple physical arrays is beneficial for computation, but increases the likelihood of overfitting - a common challenge in machine-learning. One way to avoid this is to increase the size of the training dataset, however, this luxury is often unavailable in real-world applications, especially in remote physical use-cases which must expensively acquire their own

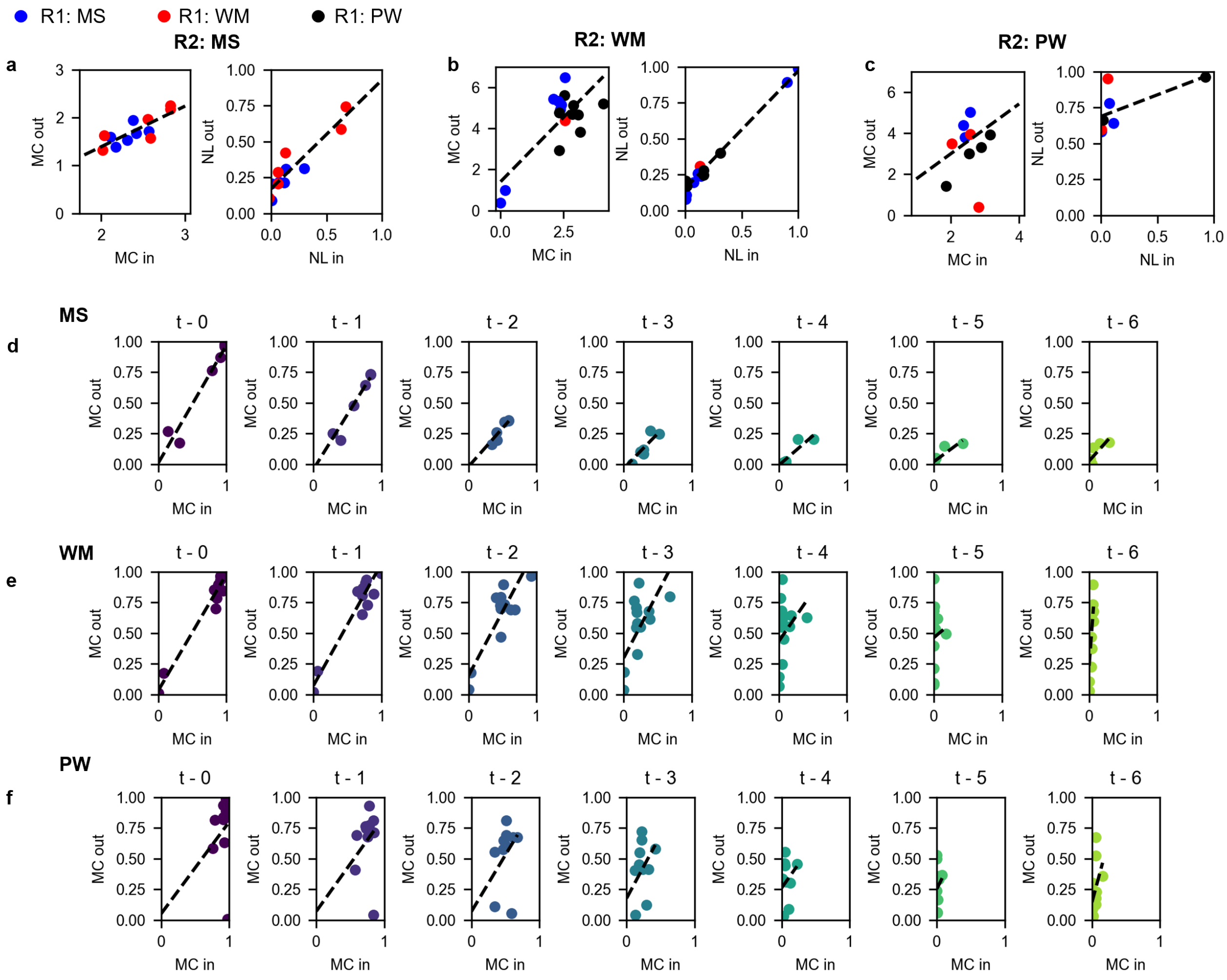

Supplementary Figure 6. Interconnection metrics and memory amplification. Memory-capacity of reservoir 1 (MC_{in}) vs Memory-capacity of reservoir 2 (MC_{out})=when R2 is a) MS, b) WM and c) PW. High memory-capacity and nonlinearity are achieved when the interconnection memory-capacity and nonlinearity are high. d-f) MC_{in} vs MC_{out} when evaluating memory-capacity on specific previous inputs from t-0 (current input) to t-6. Dashed line represents a linear fit.

data using sensors. In response, we implement a feature selection scheme[11,13] to reduce the number of network outputs by discarding less useful channels, avoiding overfitting and providing robust, accurate performance (see Methods).

Schematics of the parallel and series networks are shown in Supplementary Figure 7 a) and b) respectively. In parallel networks, data is input into multiple arrays and the FMR response of each array measured. During offline training, the response of different arrays is concatenated to give the network output (i.e. two arrays with 300 frequency output channels produce a network with 600 outputs/parameters).

In series networks, data is input to the first array/reservoir (R1) and FMR response is measured. The 300-dimensional R1 FMR response now must be converted to a 1D field input for the second reservoir array R2. To accomplish this, we take the amplitude of a specific FMR frequency-channel and map it to an input field sequence for R2. The R1 output frequency-channel is selected via per-channel memory-capacity and nonlinearity evaluation. This analysis and scaling step is performed offline, with future prospects for on-chip implementations. The FMR spectra of R1 and R2 are then combined for offline training and prediction[11].

Parallel networks are capable of enhancing performance at tasks demanding high nonlinearity and low memory. Example transformations are shown in Supplementary Figure 7 e) for $\sin(3x) + \cos(x)$ and $|\sin(x/2)|$ where improvements up to 4.4× vs. the best single array observed when combining all three arrays in parallel (further transforms are discussed later).

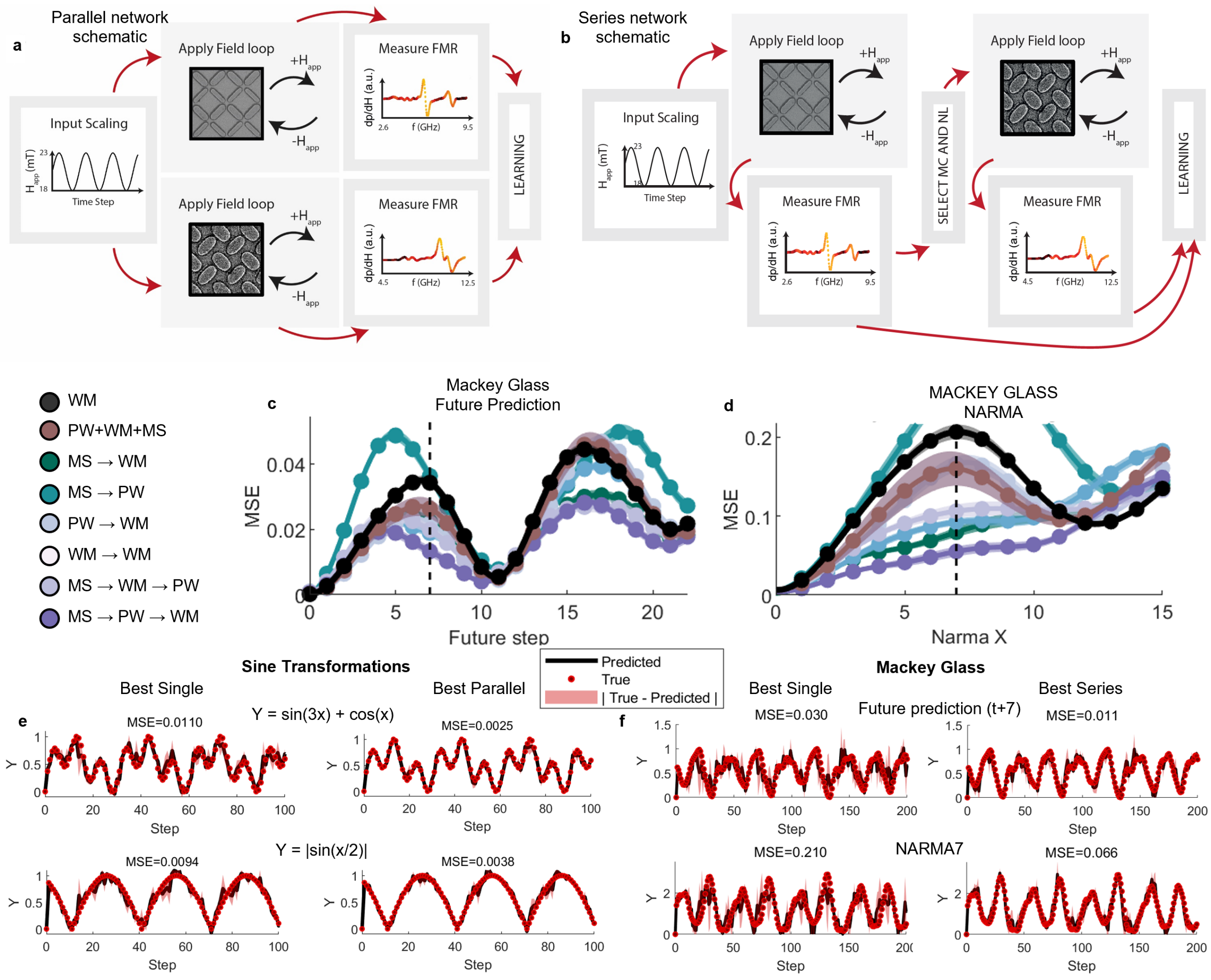

Supplementary Figure 7. Interconnecting physical reservoirs. a) Schematic of the parallel network architecture. Data is input to two separate reservoirs in parallel and their FMR spectra are combined for offline training. b) Schematic of a series network architecture. Specific output-channels from the first reservoir are input into the second reservoir. Outputs of both reservoirs are combined for offline training. c) Mackey-Glass future prediction and d) NARMA-transformed Mackey-Glass for the best series network (WM, black), best parallel network (PW+WM+MS, brown) and each series network architecture. Series networks go from low memory-capacity to high memory-capacity reservoirs. Parallel networks only marginally reduce error. MSE profiles are flattened in series networks, with improvements up to 4 $\times$. e) Example predictions for sine-transformations for the best single and best parallel network. f) Example predictions are shown for t+7 and NARMA7 for the best single and series networks.

Supplementary Figure 7 c,d) shows series network MSE vs t for future prediction (c) and NARMA-transformation (d) of Mackey-Glass time-series. The best single reservoir (WM, black) parallel network (PW + WM + MS, brown) are also shown. Parallel networks do not show significant enhancement due to the lack of memory-transfer in this architecture. Low memory-capacity (R1) to high memory-capacity (R2/R3) series network architectures significantly improve performance, up to 2.7 $\times$ and 4 $\times$ in three-layer deep networks for challenging t+7 and NARMA7 tasks respectively with predictions shown in Supplementary Figure 7 i). As in the brain, reservoir ordering is critical[11], with high memory-capacity to low memory-capacity network architectures showing weak improvements of <1.5 for all prediction tasks (supplementary note 7 discusses deep architecture ordering). Three-layer deep networks substantially outperform two layers, highlighting the benefits of more complex networks.

The best prediction is found for MS -> PW -> WM (3 layers, memory-capacity = 6.7, nonlinearity = 0.75). While the 3-layer deep network has a memory-capacity improvement of 1.31 vs. the best single reservoir, MSE values are up to 3.7× lower than the single WM sample for future prediction tasks due to the expanded range of temporal responses/timescales in the deep network. This expanded range of temporal responses results in strong flattening of MSE periodicity vs. single and parallel reservoirs, especially evident in the NARMA-transform task (f). This enhanced temporal richness is not accurately reflected in a single memory-capacity/memory-capacity score. Memory-capacity and nonlinearity metrics are valuable guides for basic reservoir evaluation, but lack the granularity for accurately predicting performance.

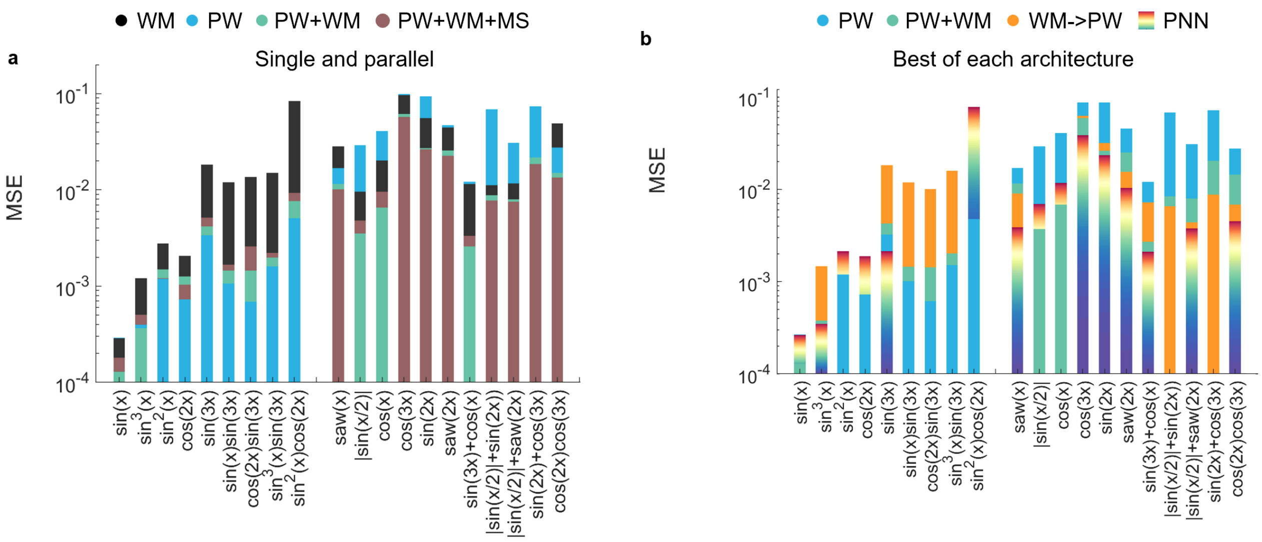

Supplementary Figure 8. Sine transformations with interconnected networks. a) MSE for single and parallel configurations when transforming a sine input. Improvements are obtained across asymmetric (memory-capacity + nonlinearity) tasks, up to 4.4 × for sin(3x) + cos(x). Performance often worsens for symmetric (nonlinearity only) tasks. b) MSE profiles the best single, parallel, series and PNN networks when transforming a sine input. Series improvements are only observed when connecting WM and PW for more complex asymmetric tasks. PNN outperforms other architectures for 9/20 tasks.

Supplementary Figure 8 a) shows MSEs of the best single and parallel networks for each signal-transformation task. Only the best 2- and 3-layer parallel architectures are shown. Lower MSE is observed for parallel architectures for asymmetric tasks requiring memory-capacity and nonlinearity. Parallel networks are able to harness memory-capacity and nonlinearity characteristics from different artificial spin reservoirs to reduce MSE for asymmetric transforms. Lowest MSEs are observed when combining WM + PW, or all three artificial spin reservoirs with performance gains up to 4.4 × for more complex asymmetric tasks. For some tasks, adding MS to create a 3 layered parallel architecture worsens performance due to the limited transformation capabilities of that reservoir replacing more useful outputs from the other artificial spin reservoirs.

Interestingly, the best parallel architectures have lower nonlinearity than the single PW (0.65 vs. 0.75). The feature selection process optimises outputs across all tasks. Introducing high memory-capacity outputs from WM to improve asymmetric tasks comes at the cost of sacrificing high nonlinearity outputs from PW, reducing nonlinearity-only task performance.

Supplementary Figure 8 b) shows MSE values for single, parallel and series networks for signal-transformation. Series networks show enhanced performance for complex asymmetric tasks requiring both MC and NL (e.g. sin(2x) + cos(3x)). For simpler symmetric and asymmetric tasks, single and parallel networks dominate.

Supplementary Figure 9 shows the future prediction and NARMA transformation performance in a deep network when going from a reservoir with high memory-capacity to a reservoir to low memory-capacity. Low improvements are observed throughout. The initial reservoir response obscures short term information. The second reservoir simply mimics this response. As such, short term information is lost.

The ordering of artificial spin reservoir's plays an important role: if R1 has low memory-capacity, its response is captures short term behaviour. When this information is transferred, a high memory-capacity R2 receives information about short-term behaviour which it can retain for longer. As such, short-term memory is retained in R1 and long-term memory is retained in R2. For high-to-low memory-capacity, R1's output will be obscured by history-dependence, limiting the amount of information retained about short term behaviour. When this information is transferred, R2 simply mimics this information. As such, neither

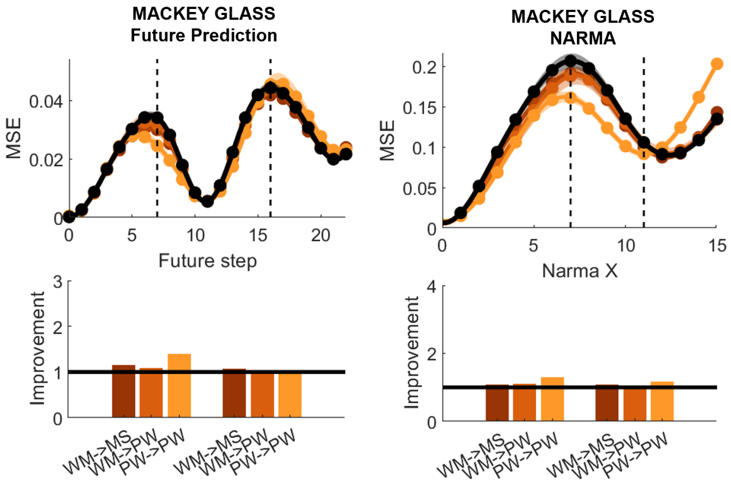

Supplementary Figure 9. Reservoir ordering effects on prediction performance. Mackey-Glass future prediction and NARMA transformation in a deep network when going from a reservoir with high memory-capacity to a reservoir with low memory-capacity. Low improvements are seen across all tasks.

R1 or R2 retain a reasonable short-term memory, only long-term correlations are present, reducing the overall memory-capacity.

Complex memory-capacity + nonlinearity signal-transformations require higher harmonic generation and responses shifted in phase w.r.t the input. By going from a high memory-capacity reservoir (WM) to a high nonlinearity reservoir (nonlinearity), both a linear and nonlinear representation of the input signal is present in the overall network (linear from high memory-capacity, nonlinear from high nonlinearity) producing a diverse set of lagged responses and higher harmonics.

Supplementary note 7 - Mackey-Glass attractor reconstruction

We use another methodology to understand the efficacy of our method in reconstructing the original time-series, even in the case of the prediction task. In Supplementary Figure 10 we plot show two prediction tasks (t+1 and t+7) for the Mackey-Glass time series (top curves). We see that at low prediction windows, both a single reservoir and a network of reservoirs reconstruct the time series. We can analyse the efficacy of the task by looking at the reconstructed attractors using the State Space Reconstructor method[14–16], embedding it in 3-dimensions with a delay of $T = 16$. We compare the original reconstructed attractor (black curves) versus the single (green curves) and PNN models (red curves). These plots contain much more information than MSE (center middle plots). As we can see, at low prediction windows (t+1) the reconstructed attractor is well approximated by both the RC models. However, at longer prediction windows (t+7) the PNN follows more closely the attractor. This can be quantified by looking at the multi-dimensional variance of the 3-dimensional time series obtained by subtracting the trajectory of the attractor from the original time-series versus the two reconstructed ones (bottom plots). We see error is much more spread at longer prediction windows for the single RC.

Supplementary note 8 - PNN in the underparameterised regime

In Figure 2 of the main text, the featuer selection algorithm is allowed to freely chose between all outputs. The total number of outputs used for each architecture is: WM - 34, MS+PW+WM - 44, MS$\rightarrow$WM - 42, MS$\rightarrow$WM$\rightarrow$PW - 93 and PNN - 13567. The enriched readout dimensionality is a distinct advantage of the PNN in terms of computational performance.

Supplementary Figure 11 shows the performance when the PNN is constrained to have a similar number of outputs the other networks. Here, 97 PNN are used during training. We find that performance is at a similar level to the three-series network. Performance is worse for some tasks. This is because the feature selection algorithm is stochastic and requires more iterations to explore the entire readout space. Additionally, the algorithm finds a set of outputs that performs well at all tasks. Given enough iterations, the performance of the PNN will at least match other architectures, as the PNN contains outputs from all other network architectures.

Supplementary note 9 - Software comparison

We now compare the performance of the various network architectures to software models, specifically echo state networks (ESN) and multilayer perceptrons (MLP). For ESN, we vary the number of internal nodes (N_{nodes}) and for each node we

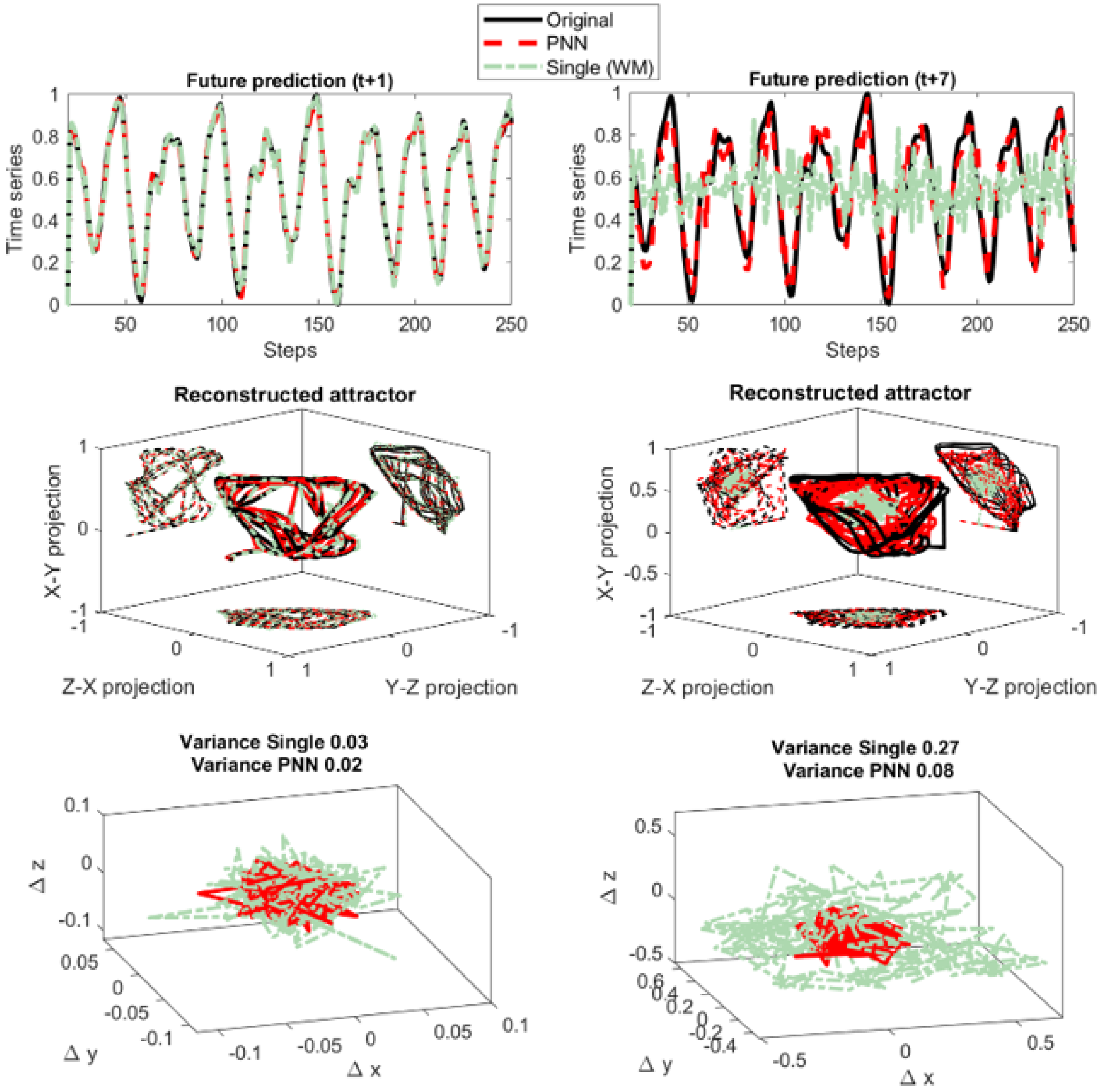

Supplementary Figure 10. Mackey-Glass attractor reconstruction. Reconstruction of the Mackey-Glass attractor using state space reconstruction for a prediction window of 1 and 7 time steps. Top panels show the original Mackey-Glass time series versus single and PNN networks. We visually see that at larger future prediction steps the combined reservoirs perform better. This can also be quantified in the reconstructed attractor (middle panels) via State Space Reconstruction, with a delay of 16 points and an embedding dimension 3, for the single and combined reservoirs. The difference between the combined and single attractor trajectories and the reconstructed attractor for the original time series is shown in the bottom right figure. We quantify the difference using the metric $v = \sqrt{varx + vary + varz}/\sqrt{3}$.

randomly initialise 50 networks as described in the Methods section *ESN Comparison*. For MLPs, we vary the size of the hidden layers (N_{hidden}) from 1-500 and number of previous inputs the MLP receives (T_{seq}) from 1-10 (see Methods for MLP details).

Supplementary Figure 12 shows the performance of ESNs with varying number of nodes (N_{nodes}) for a) Mackey-Glass future prediction, b) NARMA transform, c) NARMA transform plus future prediction as presented in the main text. Panels d-f)

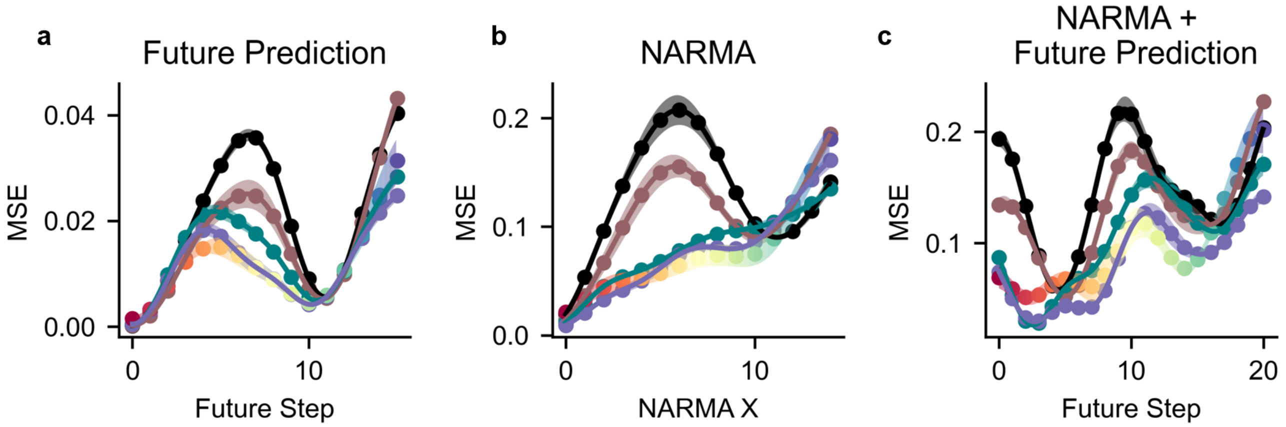

Supplementary Figure 11. Underparameterised PNN performance. MSE profiles for a) Mackey-Glass future prediction, b) NARMA transformation and c) future prediction of NARMA-7 processed Mackey-Glass for the best single, parallel, series and PNN when the PNN is forced to be underparameterised (i.e. the number of parameters is less than the size of the training set). PNN MSE is similar to the series networks as it is not able to harness all of the outputs.

show the ESN performance when varying N_{train} and N_{nodes} for each task. In a-c), the blue line represents the average error of 50 randomly initialised ESNs for a given number of internal nodes with shaded area representing $\pm 2\theta$. Coloured lines show the performance of the various networks explored in the main text. In a-c), N_{train} = 200. MSE represents the average performance over multiple tasks: t+0 to t+12 for future prediction (a), NARMA 0 - 12 for NARMA transforms (b) and t+0 to t+20 for NARMA plus future prediction (c). For ESNs, as N_{nodes} and N_{train} increases, MSE reduces as expected. For each task, we find that single arrays are matched by ESNs with 20 - 40 nodes. As the physical network complexity increases, the corresponding ESN size to match performance also increases. The ESN size required to match the PNN performance is 100 for the NARMA task and >500 for prediction tasks.

We now compare the physical network performance to MLPs. MLPs are static, i.e. they hold no information about previous inputs and will fail at predictive tasks. As such, we vary the number of previous inputs given to the MLP during each time step. Supplementary Figure 13 shows the performance of MLPs when varying the size of the hidden layers (N_{hidden}) and the number of previous inputs provided to the MLP (T_{seg}) for a) Mackey-Glass future prediction, b) NARMA transform, c) NARMA transform plus future prediction as presented in the main text. As with the ESN, MSE represents the average performance over multiple tasks: t+0 to t+12 for future prediction (a), NARMA 0 - 12 for NARMA transforms (b) and t+0 to t+20 for NARMA plus future prediction (c). The MLP MSE reduces as N_{hidden} and T_{seg} increases as expected. For T_{seg} = 1, the physical networks outperform all trialled MLPs due to the lack of past information in the MLP. We find that series networks are well matched to MLPs with T_{seg} = 2 and $N_{hidden} \sim 10$ and the PNN is matched to an MLP with T_{seg} = 3 and $N_{hidden} \sim$ 10-50 for all tasks. Whilst the resulting MLP is simple, the network interconnections must be trained using gradient descent. This is fairly inexpensive for small networks, but as network and task complexity increases, so does the expense of training MLP weights.

We now implement the same networking methodology described in the main text with software echo-state networks.

We begin by initializing ESNs with 200 nodes (to ensure a similar number of output channels in comparison to nanomagnetic arrays) and select three which display similar memory-capacity and non-linearity to the three nanomagnetic arrays. Supplementary Figure 14 shows the memory-capacity and non-linearity of the three nanomagnetic arrays alongside three ESNs with similar properties (red circles). The ESNs were found via randomly varying the hyperparameters of the ESN and selecting ESNs which had the closest characteristics.

We find that, despite the ESNs and nanomagnetic arrays displaying similar metrics, the performance shows different characteristics. For the two high memory-capacity ESNs, the MSE rises slightly from future step 1 – 3, and then becomes flat, whereas the hardware reservoirs display a periodic profile in the MSE. We find that it is challenging to find an ESN that displays the same characteristics as the hardware reservoirs. This is not surprising, as nanomagnetic reservoirs and software ESNs are governed by different underlying dynamics. In nanomagnetic arrays, coupling arises from the dipolar field which decays spatially and hence coupling only occurs locally, whereas in ESNs, coupling is randomly assigned across the network. In ESNs, the coupling strengths between nodes are initialised and remain fixed, whereas in nanomagnetic arrays, the coupling between neighbouring elements depends on the state of those elements (e.g. uniform magnetisation or vortex). Furthermore, in ESNs, only certain nodes are coupled to the input, which enhances memory-capacity as certain nodes require a number of

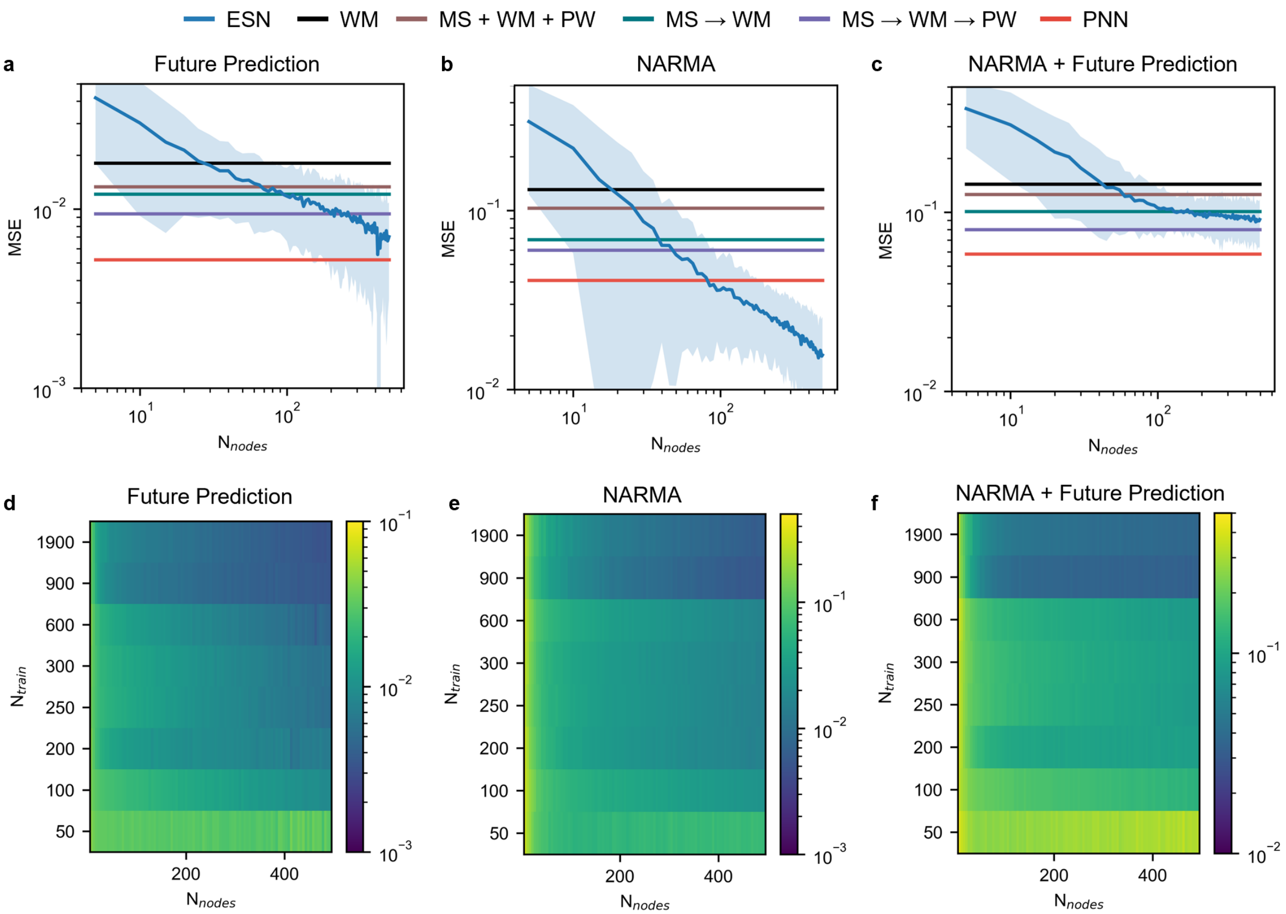

Supplementary Figure 12. **Comparison to echo state networks.** ESN performance when varying the number of ESN nodes for a) Mackey-Glass future prediction, b) NARMA transform, c) NARMA transform plus future prediction as presented in the main text. In each plot, the blue line and shaded region represent the average MSE and error over 50 randomly initialise ESNs at a given number of nodes (N_{nodes}). Solid flat lines represent the MSE from the physical networks presented in the main text. Here, the training data size (N_{train}) = 200. MSE is an average over multiple tasks: t+0 to t+12 for future prediction (a), NARMA 0 - 12 for NARMA transforms (b) and t+0 to t+20 for NARMA plus future prediction (c). Panels d-f) show heatmaps of MSE for varying N_{train} and N_{nodes}.

network updates before receiving information about a particular input. On the other hand, in nanomagnetic arrays, each node is subject to the input data when it is first applied. Finally, experimental nanomagnetic systems are subject to noise, whereas ESNs are noise free. It is possible that, with extensive optimisation, an ESN could be initialised that has more similar characteristics to nanomagnetic arrays, however, achieving this is beyond the scope of this report.

We note that when initialising ESNs, the majority of them had memory-capacity far beyond the nanomagnetic arrays (close to 8).

We now interconnect these arrays following the same methodology used to connect different nanomagnetic arrays. We begin by analysing the channel specific time correlation and non-linearity of the three ESNs displayed in Supplementary Figure 15.

When comparing these results to the nanomagnetic reservoirs (SI Supplementary Figure 5) we again see fundamental differences in the dynamics of ESNs vs nanomagnetic hardware. For the low memory-capacity ESN (ESN 1), we find that all channels are only correlated with short-term previous inputs. Conversely, for the higher memory-capacity ESNs (ESN 2&3), there are a broad range of correlations. Some channels are correlated with short term previous inputs, whereas some are correlated with previous inputs many time-steps ago. In contrast, in nanomagnetic arrays, all of channels are most strongly correlated with short-term inputs and only a handful are correlated with long-term previous inputs. This is a symptom of the

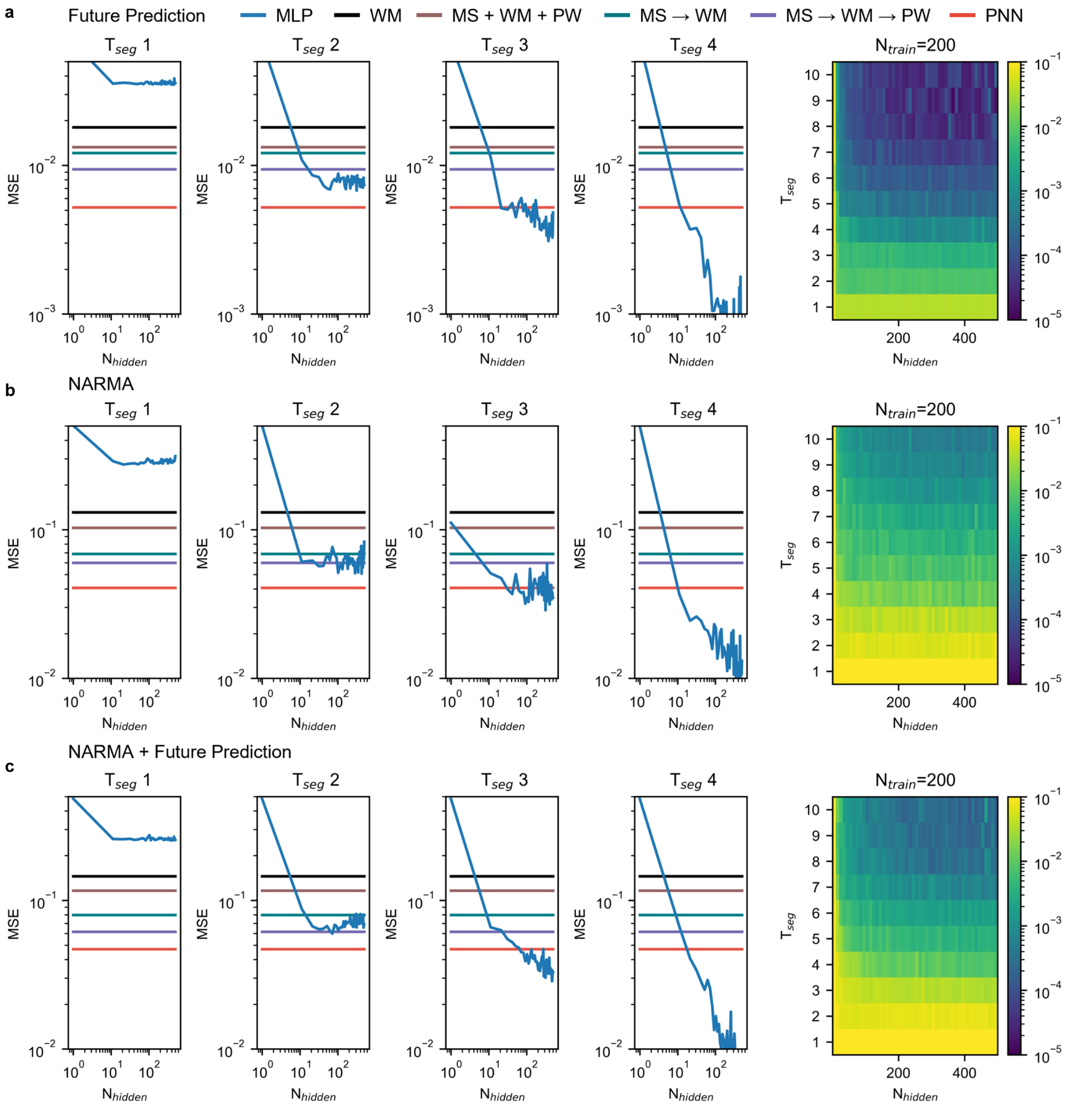

Supplementary Figure 13. Comparison to mulitlayer perceptrons (MLP). MLP performance when varying the size the hidden layers (N_{hidden}) and the number of previous inputs given to the model during training (T_{seg}) for a) Mackey-Glass future prediction, b) NARMA transform, c) NARMA transform plus future prediction as presented in the main text. In each plot the blue line represents the MSE of the MLP for a given number of hidden nodes. Solid flat lines represent the MSE from the physical networks presented in the main text. Here, the training data size (N_{train}) = 200. MSE is an average over multiple tasks: t+0 to t+12 for future prediction (a), NARMA 0 - 12 for NARMA transforms (b) and t+0 to t+20 for NARMA plus future prediction (c).

differences between internode couplings in ESN when compared to nanomagnetic arrays. The random, sparse connections in ESN allow many different dynamic timescales whereas the spatially constricted, local couplings in nanomagnetic systems prevent this behaviour.

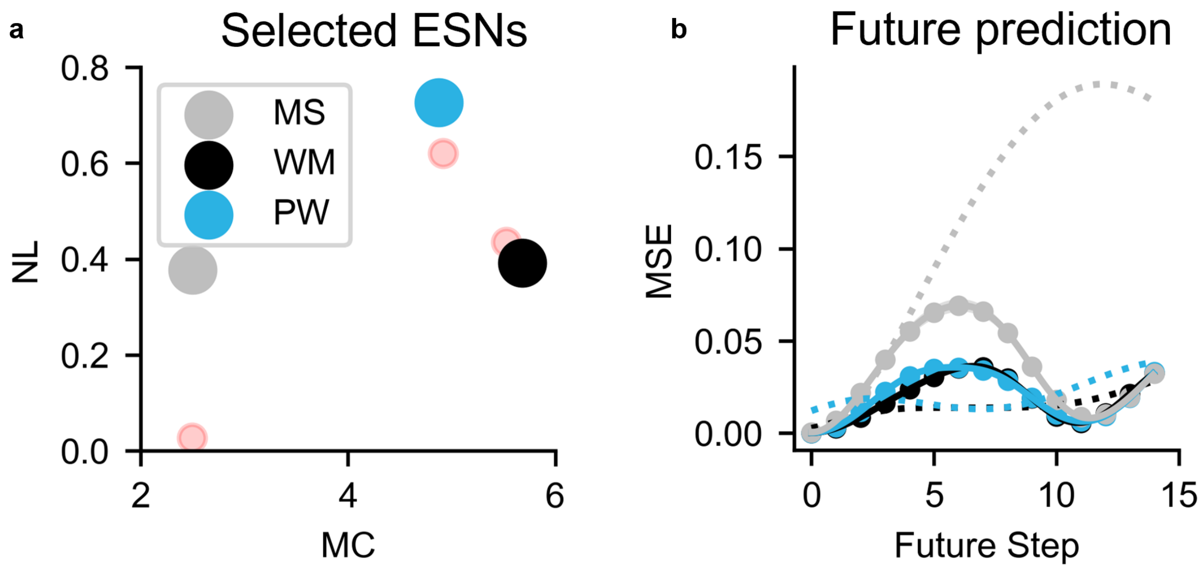

Supplementary Figure 14. Metrics and MSE of echo-state networks. a) Memory-capacity (MC) and non-linearity (NL) of the nanomagnetic arrays and three ESNs (red markers) chosen to have similar metrics. b) Performance when predicting future values of the Mackey-Glass equation for nanomagnetic arrays (solid line) and ESNs with similar metrics (dotted lines).

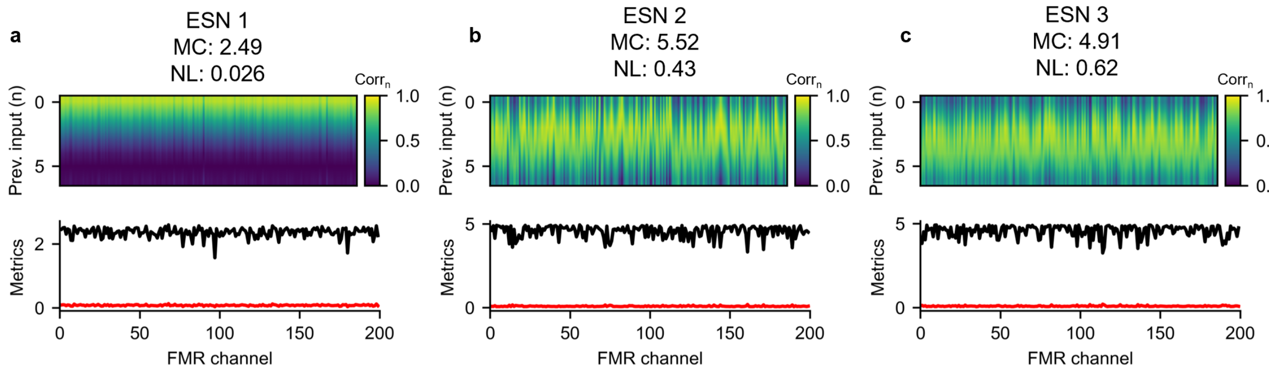

Supplementary Figure 15. ESN metrics. Per-channel correlation and non-linearity for the three selected ESNs are shown in a-c.

For each ESN, we now select six channels: three with the highest memory capacity and three with the highest non-linearity, giving a total of 18 input time-series to feed into the next layer. Each ESN is the subject to all input sequences to produce 54 2-series networks. We then create 3-series networks by selecting the 2-series architectures which: 1) begin with the low memory-capacity reservoir (ESN 1) and 2) which display the lowest MSE. We then perform the same per-channel metric analysis, generating 9 input sequences which are passed to the final ESN to form a 3-series network. Finally, we combine the responses from all single, 2-series and 3-series networks using the same methodology as the PNN. We evaluate the performance of the ESN networks using the same feature selection methodology described in the Methods section of the manuscript.

Supplementary Figure 16 compares the performance of single, 2-series, 3-series and PNN architectures for the three prediction tasks presented in the main text. The top row shows the nanomagnet response previously included in the manuscript and the bottom row shows the ESN network results. The ESN results displayed are taken from the architecture with the lowest MSE over all future / NARMA steps for a given task. When interconnecting ESNs, we find that whilst improvements are observed for some tasks, they are less pronounced than for the physical system. Series networks only show improvements for

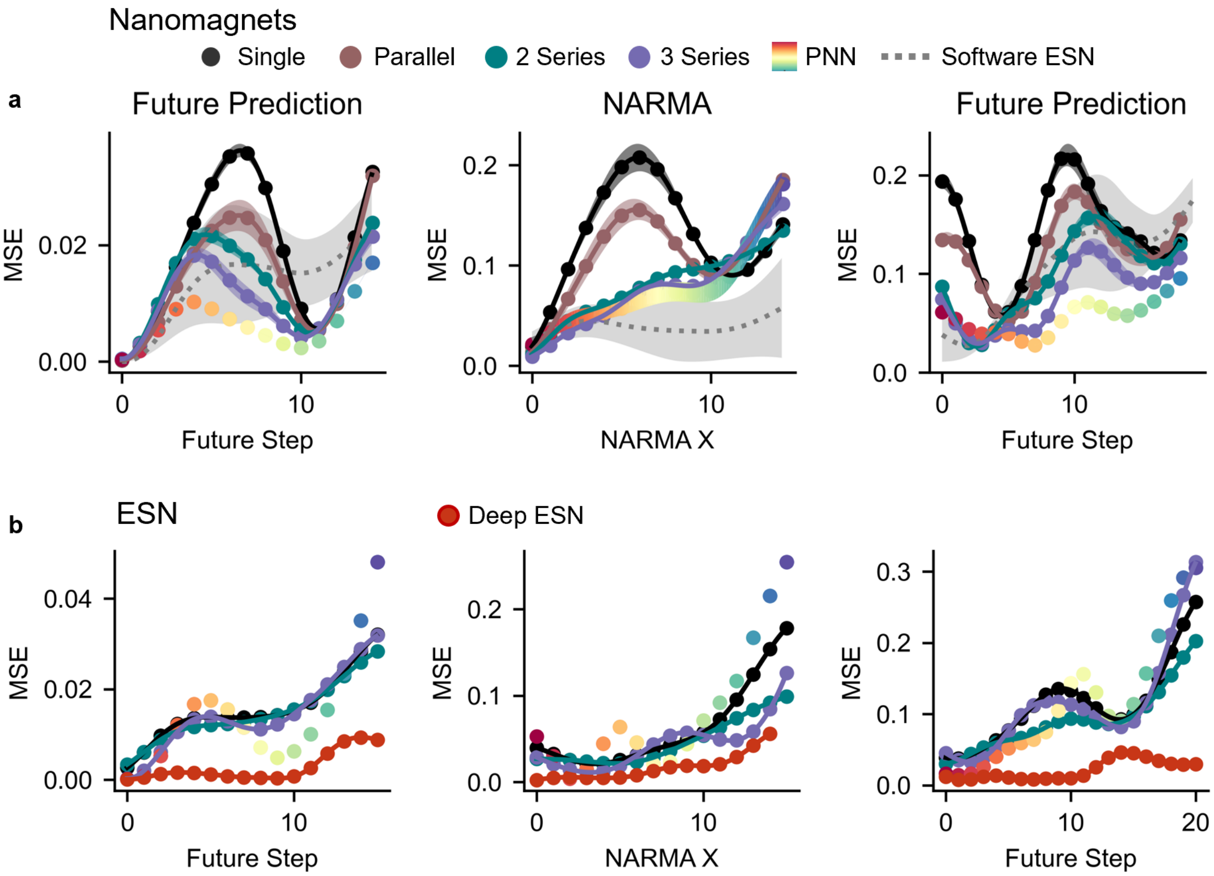

Supplementary Figure 16. Comparison between nanomagnetic arrays and software ESNs when interconnecting networks. a) shows the experimental arrays from the main manuscript. b) shows the ESN networks.

future prediction t+1 and t+2, NARMA 10 and above, and NARMA + Future prediction t+4 to t+13, whereas series networks improve all predictions for the nanomagnetic arrays. Software analogues of the PNN architecture show improvements for more tasks (notable future prediction t+7 – t+12), but also have worse performance for other tasks. We believe this is because single ESNs already display a rich set of memory time-scales, and hence interconnecting arrays does not enrich the overall network to the same extent. These results give us an indication that interconnecting physical systems can give MSE improvements which produce a similar level of performance to software counterparts. However, we stress here that the initial ESNs selected are not optimised for performance. If we were to optimise ESNs for performance, then it is likely that the software network would outperform nanomagnetic hardware.

In addition, we test deep echo state networks, where multiple interconnections between different ESN layers are made (i.e. multiple nodes from one ESN connect to multiple nodes in another) in a similar manner to previous work of some of our authors[11]. We connect the three ESNs with similar characteristics to the physical nanomagnetic reservoirs into a 3 x 3 network, whereby each ESN is present in each layer. Layer interconnections are initialised randomly and we average over 10 random trials. The prediction results for the three tasks are shown in Supplementary Figure 16 (red curve, ‘Deep ESN’). As expected, the performance dramatically improves compared to our proposed method as there is an increase in the amount of information being transferred between different layers. Currently, such interconnections are not possible with the hardware approach as each input is only able to accept one input per time step. If a physical system is used which can take multiple inputs at each time step (e.g. memristors), we can connect it in this way and achieve a computationally powerful network with just a handful of nodes.

Supplementary note 10 - Further overparameterisation details

We now provide further details regarding the overparameterisation regime, specifically, how train length, network sub components and task affect the ability to reach a beneficial overparamterised regime.

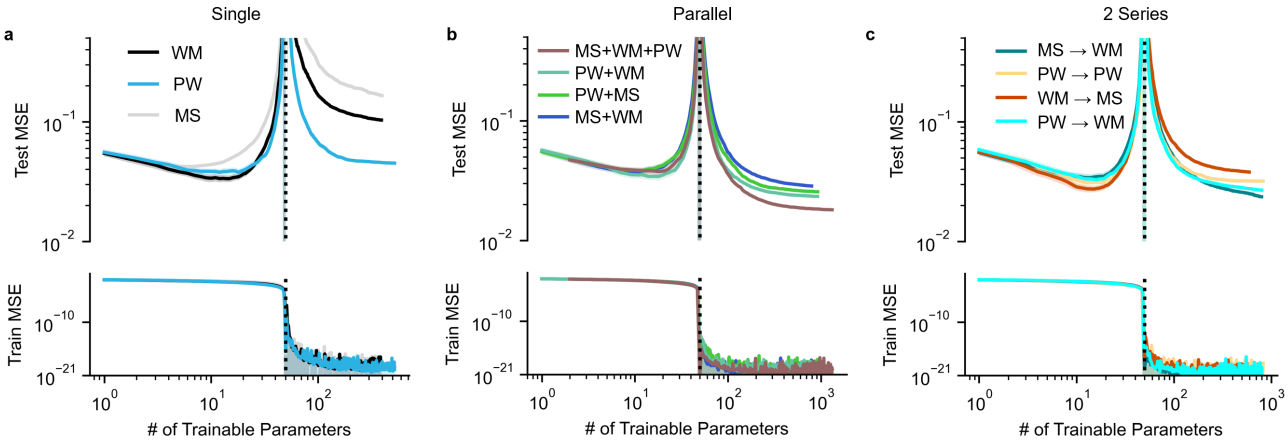

Supplementary Figure 17. Overparameterisation with all PNN architectures. MSE dependence on the number of trainable parameters for a) Single networks, b) Parallel networks and c) 2-series networks

We begin with a discussion of how the network subcomponents and architecture affect the effective dimensionality of the output and therefore ability to reach a beneficial overparameterised regime. Supplementary Figure 17 shows the dependence of the number of trainable parameters (i.e. number of output channels used during regression) on the MSE for a) single, b) parallel and c) 2-series networks. Here, MSE is an average over 6 prediction tasks (see methods for details) encompassing tasks that all networks perform well and those which simpler networks struggle. Beginning with the single arrays, we see that no single array benefits from being in an overparameterised regime as the minimum MSE in this region is higher than the minimum MSE in the underparameterised regime. However, we see that the underlying array affects the ratio of MSE_{OP} to MSE_{UP}. PW shows a far greater reduction in the overparameterised regime compared to WM and MS. Combining these results with the memory capacity and non-linearity calculations in and the spectral evolution during computation in Figure 1 of the main text, we see that having a diverse spectral output is key to achieving a beneficial overparameterised regime. PW has structural diversity, producing a large variety of magnetic states which are detected at different resonant frequencies. MS is a highly linear reservoir, with relatively simple dynamics and magnetic elements which all occupy the same frequency space. WM is a linear sample with strong memory capacity, with magnetic elements largely occupying the same frequency space, but behaving in a complex manner. For an equivalent frequency range and resolution, PW fills out the frequency space, producing a diverse set of spectral outputs. When in the overparameterised regime, the regression can harness this diverse response to produce a lower MSE in comparison with the spectrally limited WM and MS arrays.

Moving to parallel networks, we see that all combinations of parallel networks benefit from an overparameterised regime. This is expected when considering the effective dimensionality of the parallel network outputs. Each of the three networks has a distinct set of internal dynamics and output spectra, as such, combining them in parallel enhances the spectral diversity, enabling networks to benefit from an overparameterised regime. Comparing the parallel networks with different architectures, we see that the improvements gained depend on which networks are included. Combining the two low non-linearity reservoirs (MS+WM) shows a weaker improvement than combining creating a 2 parallel network with the non-linear PW sample as expected. For series networks, the level of improvements depend on the arrangement of nodes as seen in the underparameterised regime. WM->MS shows worse overparameterised performance than MS→WM despite the theoretical internal dynamics being the same for each network. As we are assessing performance on a prediction task, both the separated node dynamics and combined dynamics of the system plays a role. MS→WM is more capable of transferring previous information and increasing memory capacity, leading to better predictions and a lower MSE. As such, both the range of dynamics of each node, and there arrangement play a role. Interestingly, WM→MS displays better improvements than when combining MS and WM in parallel. Here, the internal dynamics are the same and the difference arises from the input that the second layer receives. By feeding the output of one array to the input of the next, an additional set of diverse dynamics arises from the input sequence.

Supplementary Figure 18 a) shows the double descent curves for three series networks MS→MS, WM→WM and PW→PW. As mentioned, MS is a low non-linearity, low memory capacity reservoir. The readout diversity for this array is low. When creating a 2 series network with MS in both layers MSE OP is higher than MSE UP as the readout diversity is not sufficient to

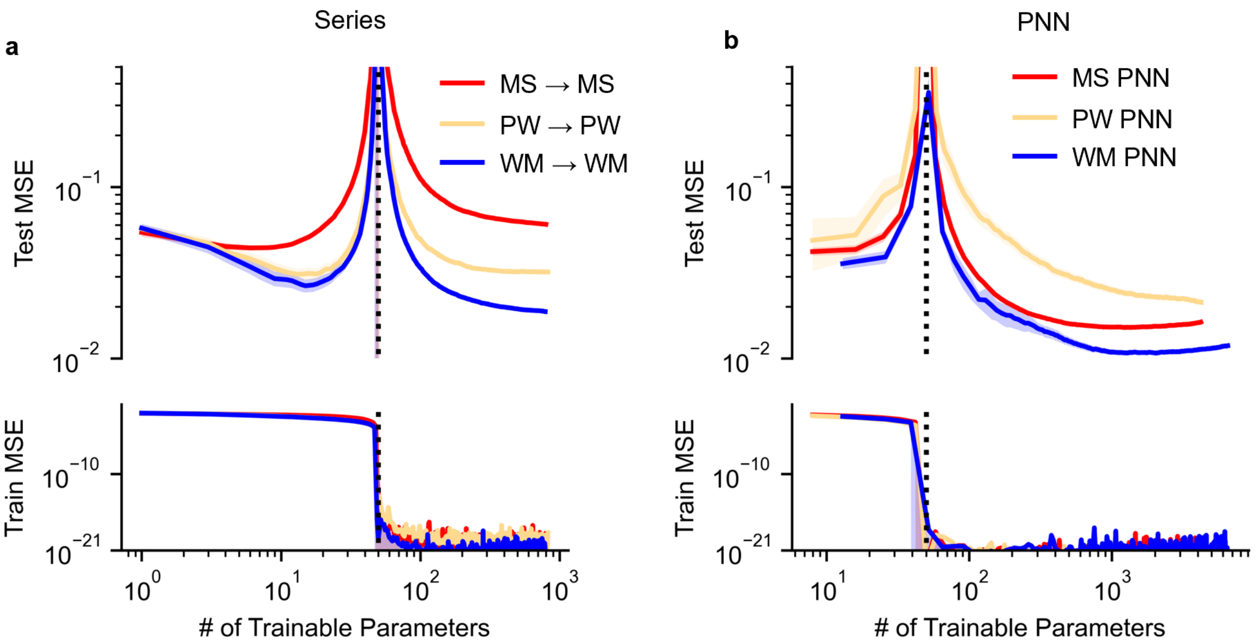

Supplementary Figure 18. Single array overparameterisation. MSE dependence on the number of trainable parameters for network with one physical system. a) MSE curve for 2-series networks comprising one physical system for each reservoir explored in this work. b) MSE curve for physical neural networks comprising one physical system. For 2 series networks, only WM→WM reaches a beneficial overparameterised regime. For PNNs, all systems can reach a beneficial overparameterised regime.

overcome overfitting. Creating series networks with systems that have more complex behaviour and higher readout diversity can lead to a beneficial overparameterised regime. As such, by designing an appropriate system, one can reach overparameterisation with just a single 2 series network. Note that if we combined two copies of a node in parallel, the results would be equivalent to a single system as the two sets of outputs would be identical. Figure X b shows the double descent curve when creating a PNN out of a single system. Here, we collate all responses from each sample from the 1st and 2nd layer of the PNN, such that a network will only contain one system as its node, but will receive a variety of different inputs. In each case, the system is able to reach overparameterisation. The implications of this are that even with a low non-linearity, low memory capacity system, one can reach a beneficial overparameterised regime by feeding multiple inputs to that system to access different dynamics. These inputs can be produced from the output of the first layer, or by masking the original input signal. Note than we can not compare the final MSE's of the networks in this plot as the inputs for a specific array were not necessarily optimised to produce a high-memory response. For example, here, PW PNN has higher MSE than MS PNN. This is due to many of the PW arrays receiving highly non-linear inputs from the first layer in order to assess how output and input metrics are related in Figure 2 e) of the main text.

Supplementary Figure 19 shows the double descent curves for selected architectures when varying the train length from 100 – 200. As N_{train} increases, the MSE in the OP regime inreases for single, series and parallel networks as the effective dimensionality of the readout is too small to overcome overfitting. The PNN strongly benefits from an overparameterised regime in all cases.

Finally, we discuss how task affects overparmeterisation. Supplementary Figure 20 shows the MSE dependence on the number of parameters when predicting a) t+5 and b) t+11 of the Mackey-Glass equation. A similar trend is observed in both cases. This is true for all future predictions evaluated (not shown). Here, whilst the task changes, the requirements of the network do not. Both tasks require strong memory capacity to achieve good performance as seen in Figure 2 of the main text.

By changing the requirements of the task, we start to see greater differences in the benefits of overparameterisation. Supplementary Figure 20 shows double descent curves transforming a sinusoidal input to c) $\sin^2(x)$, d) $\sin(3x) + \cos(x)$ and e) $\cos(2x)\cos(3x)$. Here the tasks require different levels of non-linearity and memory.

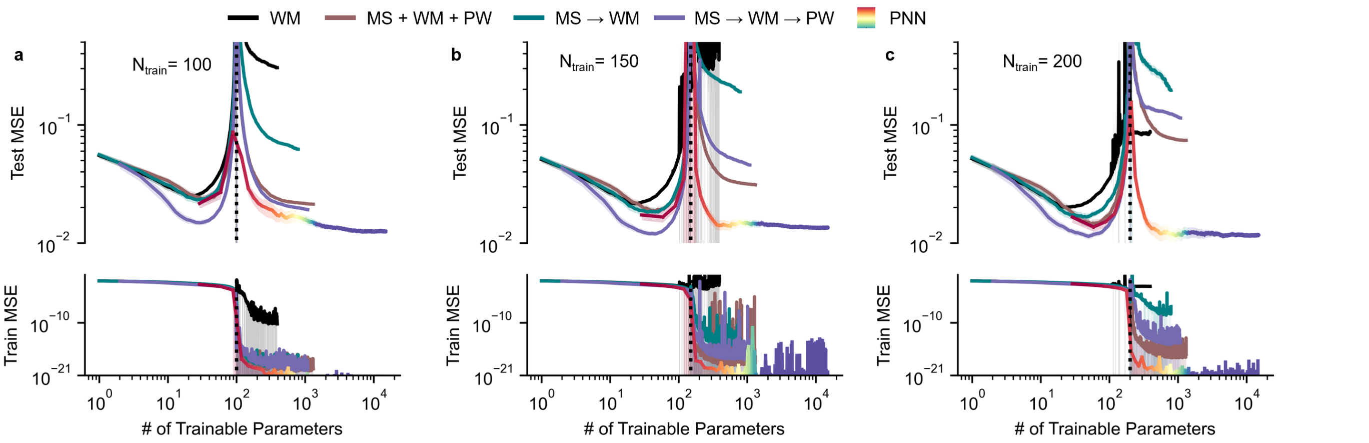

Supplementary Figure 19. MSE dependence on the number of trainable parameters when varying the size of the training set. a,b,c) are training lengths of 100, 150 and 200 respectively. For larger training dataset sizes, single, parallel and series networks do not reach a beneficial overparameterised regime due to their limited effective readout dimensionality. PNN can reach a beneficial overparameterised regime in all cases.

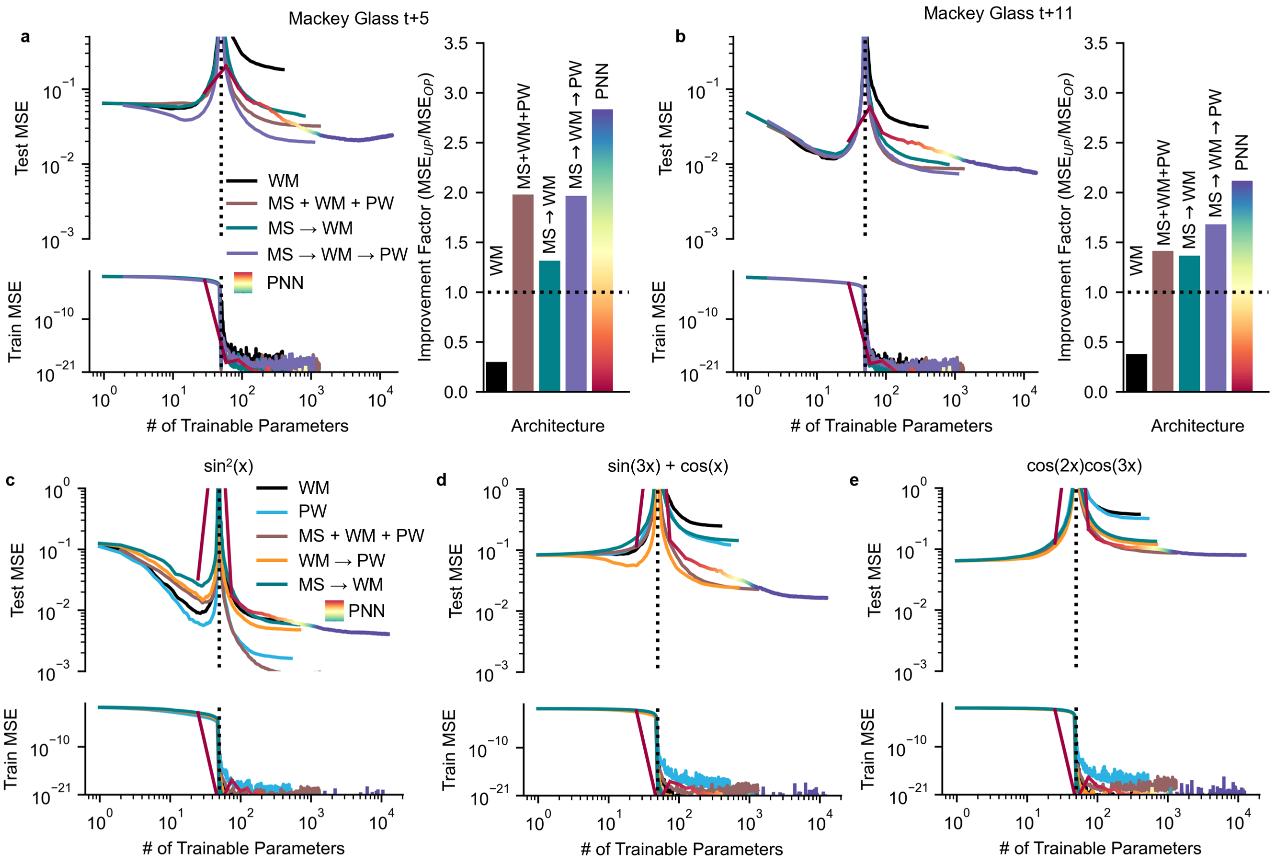

Supplementary Figure 20. Effect of task on overparameterisation. MSE dependance on number of parameters when predicting a) t+5 and b) t+11 of the Mackey-Glass equation as well as transforming a sinusoidal input to c) $\sin^2(x)$, d) $\sin(3x) + \cos(x)$ and e) $\cos(2x)\cos(3x)$. The benefits of overparameterisation are task dependent.

Supplementary note 11 - Overparameterisation with gradient descent

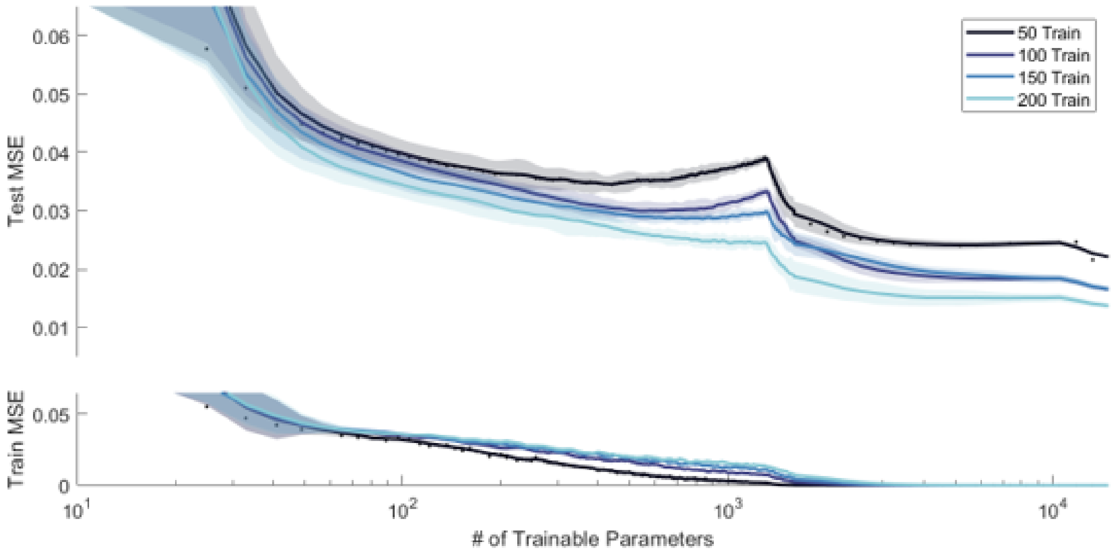

Supplementary Figure 21. Overparameterisation with gradient descent. Train and test MSE for the PNN when increasing the number of outputs and the length of the training set. Here, gradient descent for weight training. Here network weights are added by layer order, whereby outputs are added from the first PNN layer, then second layer, then final layer.

Supplementary Figure 21 shows the train and test MSE for the PNN architecture when increasing the number of parameters. Here, gradient descent is used to optimise network weights. Here, network outputs are added in the order that they appear in the PNN network i.e. outputs are added from the first layer, then the second layer, then the final layer. In the main text, outputs are sampled randomly from all layers. We observe the same qualitative trends as with linear regression, whereby the train and test MSE reduce in the underparameterised regime. Test MSE increases in the overfitting regime and then reduces in the overparameterised regime. The number of outputs required to reach an overparameterised state is higher than in the main text due the way network weights are added. This shows that the observed results are robust to the training method used, and the method of sampling outputs.

Supplementary note 12 - Further meta-learning predictions

Supplementary Figure 22 shows further examples of meta-learning predictions for a variety of targets. Both the PNN and the best single reservoir (WM) are shown. The PNN is able to accurately reproduce a wide variety of targets whereas the single reservoir fails at all tasks. As mentioned in supplementary note 4, the symmetry of the signal with respect to the input signal determines whether a signal transformation task requires non-linearity only, or memory and non-linearity. The $\sin^2(x)$ transformation is symmetric, requiring only non-linearity. In this case, we see tht all networks considered reach beneficial overparameterisation. The task is relatively simple, and effectively dimensionality required to perform well is low. Here, we see that PW and MS+WM+PW outperform the series and PNN networks. As mentioned in supplementary note 4, this is because series and PNN networks are designed to increase history-dependent response, which hinders performance for symmetric sine transforms. Both $\sin(3x) + \cos(x)$ and $\cos(2x)\cos(3x)$ are asymmetric with respect to the input, requiring the same input value to be transformed to different outputs across the waveform and therefore require both memory capacity and non-linearity. For $\sin(3x) + \cos(x)$, only PNN, parallel and WM→PW networks reach beneficial overparameterisation. For $\cos(2x)\cos(3x)$, no networks reach beneficial overparameterisation. As such, care must be taken to match the network architecture to the task to achieve the best performance in the overparameterised regime.

Supplementary note 13 - Performance comparison to conventional hardware

We now benchmark the resources required to run echo-state networks (the closest software analogue to physical reservoirs) and compare this to the power consumption, speed, and energy consumption of both our current computing architecture and proposed future devices.

In software, an ESN is initialised with random interconnections. Applying input and updating the ESN takes the form of a set of matrix calculations as:

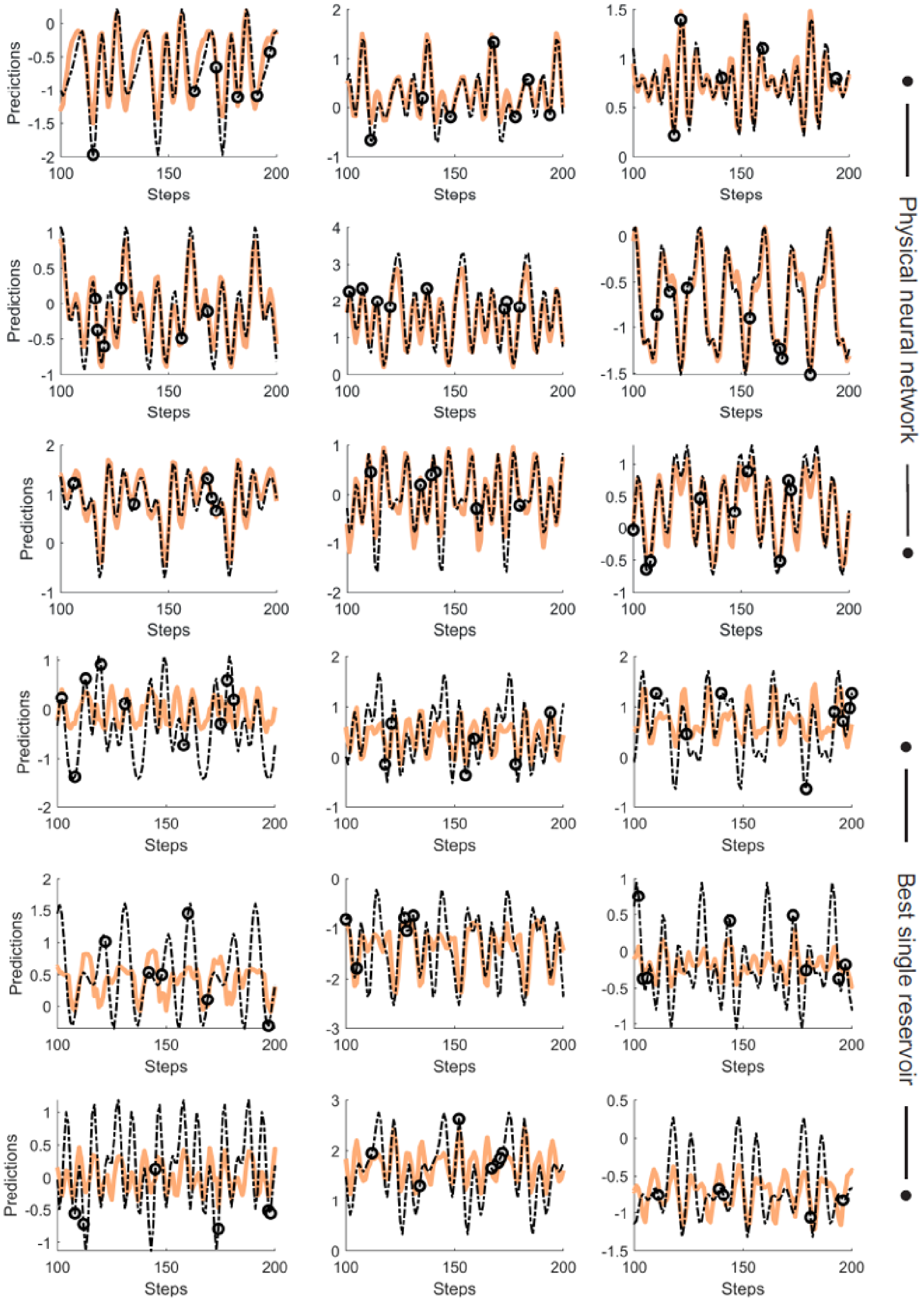

Supplementary Figure 22. Few-shot learning examples. Further examples of frequency decomposition task with the PNN and comparison with the best single reservoir (WM).

$$\mathbf{x}(t+\delta t) = (1-\alpha)\mathbf{x}(t) + \alpha f(\mathbf{W}_{in}\mathbf{s}(t) + \mathbf{W}_{esn}\mathbf{x}(t)) \quad (1)$$

Where $\mathbf{x}(t)$ is the ESN state, $\mathbf{W}_{in}$ and $\mathbf{W}_{esn}$ are matrices representing the randomly initialised input and internode weights, α is a scaling factor and f is a non-linear activation function (here tanh). This is repeated for the entire input sequence, with states saved at every input. Following this, regression is performed on the saved states to give a computational output.

For nanomagnetic arrays, a state update corresponds to application of an input (global magnetic field, Oersted field or spin-orbit torque). The array updates itself naturally through the physics. The state is then readout via ferromagnetic spectroscopy and saved via analogue to digital conversion. After this, regression is then performed via conventional CMOS, as it is in the software reservoir. As such, any differences in performance arise from the input / update process, the readout, and analogue to digital conversion.

We begin by analysing the resources of software reservoirs. A common way of benchmarking the resources of software algorithms is to analyse the number of floating-point operations (FLOPs). We can dissect the reservoir update formula to analyse the number of FLOPs in relation to the number of nodes in an ESN (N). For a reservoir with N nodes, $\mathbf{x}(t)$ is a matrix of size [N,1], $\mathbf{W}_{in}$ is a matrix with size [N,1], $\mathbf{W}_{esn}$ is a matrix with size [N, N], α is a float, f is a non-linear activation (here tanh). We note than the number of FLOPs for addition of two matrices of size [m,n] is *mn* and the multiplication of two matrices of size [m,n], [n,p] is *nm(2p-1)*. From this we get the theoretical number of FLOPs for each subcomponent of the update formula:

- (1-α) – 1 FLOP
- (1- α)$\mathbf{x}(t)$ – multiplication of a float and [N,1] matrix = N FLOPs
- $\mathbf{W}_{in}\mathbf{s}(t)$ – multiplication of [N,1] and [1,1] matrix = N FLOPs
- $\mathbf{W}_{esn}\mathbf{x}(t)$ – multiplication of [N,N] and [N,1] matrix = N(2N-1) FLOPs
- $\mathbf{W}_{in}\mathbf{s}(t) + \mathbf{W}_{esn}\mathbf{x}(t)$ – addition of two [N,1] matrices = N FLOPs
- f – activation function of a [N,1] matrix. tanh takes 20 FLOPs per value. = 20N FLOPs
- Final addition of two [N,1] matrices = N FLOPs

In total, to update a reservoir we have N + 2N-1 + N(2N-1) + N + 20N + N = $2N^2$ + 24N - 3 FLOPs. In practice, this value is higher due to the initialisation of a reservoir, and the optimisation of reservoir properties (e.g. running multiple random iterations to find an optimal configuration). We must also take reservoir sparsity into account (echo-state networks do not have all-to-all connections, instead they are sparse networks where only ∼20% of connections are non-zero) giving a final equation of FLOPS = $2(sN)^2$ + 24sN-3 FLOPs where s is the sparsity factor (between 0 and 1).

From this, we can benchmark the time and energy required to perform a reservoir update on a variety of different hardware. We compare the FLOPS (FLOPs per second) and power consumption of GeForce RTX 4070[17], Intel Core i7-13700H[18], Raspberry Pi-4[19], and Zynq SoC Z-7020 FPGA[20] in Supplementary Table 1. FLOPS/Watt is calculated as FLOPS / Thermal Design Power.

System	FLOPS	Thermal Design Power (W)	FLOPS / Watt
GeForce RTX 4070	4.30E+13	200	2.15E+11
Intel Core i7-13700H CPU	5.38E+11	45	1.19E+10
Raspberry Pi-4	1.35E+10	6.6	2.05E+09
Zynq SoC Z-7020	1.80E+11	2.5	7.20E+10

Supplementary Table 1. Performance of three conventional computing hardware options

We can use this information to calculate the time and energy required to update a reservoir using: Time = $FLOPs_{update}$ / FLOPS and Energy = $FLOPs_{update}$ / FLOPS/Watt where $FLOPs_{update}$ is the number of FLOPs required to update an ESN of a particular size. Supplementary Table 2 compares the time and energy required to update a 30 node and 500 node ESN for each of the above hardware with a sparsity of 0.2.

We can compare this to the time, energy and power required to input and readout data in a nanomagnetic array. Our existing system uses a NanOsc CryoFMR PPMS probe to record spectra, and hence uses a superconducting magnet to apply fields. Operating this system carries a vast energy cost. Instead, we can compare this to a similar experimental set-up that uses an electromagnet. The electromagnet supplies a magnetic field using 1 A and 5 V for 100 ms, resulting in a power of 5W and energy consumption of 0.5 J. For readout, we use a microwave source with power of 17 dBm (0.05 W), which takes ∼20s to record the entire spectra. This gives an energy of 1 J. Finally, the output is fed into a lock-in amplifier, which runs at 60 W,

System	Time (30 nodes) (s)	Energy (30 nodes) (J)	Time (500 nodes) (s)	Energy (500 nodes) (J)
GeForce RTX 4070	4.95E-12	9.91E-10	5.21E-10	1.04E-07
Intel Core i7-13700H CPU	3.96E-10	1.78E-08	4.17E-08	1.87E-06
Raspberry Pi-4	1.58E-08	1.04E-07	1.66E-06	1.09E-05
Zynq SoC Z-7020	1.18E-09	2.96E-09	1.24E-07	3.11E-07

Supplementary Table 2. Energy and time when updating an echo-state network with 30 and 500 nodes using conventional hardware.

requiring 1.2 kJ, by far the dominating factor. As such, the total power, time, and area of the existing array is 65 W, 20.1s and 1 m^2.

We now calculate the power time and energy of a projected device. Below we separate out each of the components described in the manuscript. We assume Oersted field pulses of 1 ns, spin orbit torque (SOT) switching pulse of 0.25 ns, RF power of 0 dBm (1 mW) for sequential measurement, RF noise source power of 150 mW for parallel measurement and measurement time per channel of 14 ns (steady state precession requires 100 oscillations which gives $\sim$14 ns for a frequency of 7 GHz resonance[21], for 200 channels this gives 2800 ns). Magnetic array area is based on 5 x 5 um projected device.

We compare Oersted field and spin-orbit torque input methods. To calculate the current required to produce a Oersted field from a current carrying microstrip, we use the method of Ref.[22]. The Oersted field generated in a current carrying wire is defined by Ampere's law. We assume a wire with a 10 μm width and infinite length, split into finite elements of size . A differential current dI_z at position (x_j, y_j), produces a differential magnetic field $dH_{x,y}$ described as follows:

$$dH_{x,y} = \frac{dI_z}{2\pi\left((x-x_j)^2+(y-y_j)^2\right)}\begin{pmatrix}\frac{-(y-y_j)}{(x-x_j)}\end{pmatrix} \quad (2)$$

By numerically integrating over all positions in the wire, the magnetic field at (x,y) is obtained. Supplementary Figure 23 a) shows the magnetic field profile of a wire with width 5 μm, thickness 50 nm and infinite length. Supplementary Figure 23 b) shows the x-component of the magnetic field at x = 0 nm, y = 35 nm. To generate a 25 mT field, a current of 200 mA ($8\times10^{11} Am^{-2}$) is required.

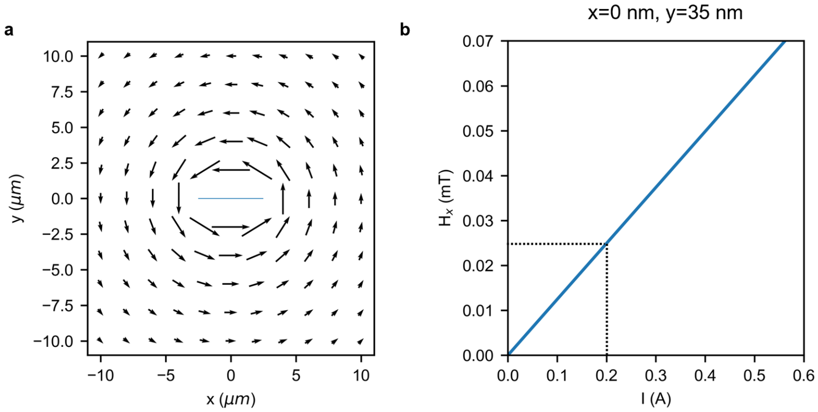

Supplementary Figure 23. Oersted field calculation. a) Oersted field profile from an infinite wire with width of 5 μm and thickness of 50 nm. The blue line represents the stripline. b) x-component of magnetic field at x = 0 nm and y = 35 nm as a function of current. Dotted lines represent the current needed to produce a 25 mT field.

Assuming the strip-line is made of Cu with a resistivity ρ = 16.8 $n\Omega m$, the resistance of a Cu strip with dimensions of 5 μm $\times$ 5 μm $\times$ 50 nm is given by R = $\rho l/A$ = 0.336 Ω. The power consumption can be calculated as P = I^2R = 13.4 mW.

To calculate the Ta power consumption for spin torque switching we follow the same methodology. For a current density of 10^{11} A m^{-2} and Ta sheet dimensions of 5 μm $\times$ 5 μm $\times$ 5 nm, we have a current of 2.5 mA. Assuming a Ta resistivity of 131 $n\Omega m$ gives a resistance R = 26.2 Ω and P = 0.164 mW.

The torque generated on a ferromagnet through spin-obit effects is inversely proportional to the thickness of the ferromagnetic material[23]. For thicker elements (20 nm) considering torques alone, we can expect at least a 10 X increase in the current density

System	Power (W)	Time (s)	Energy (J)	Dimensions (L x W)	Area (mm^2)
Oersted field (pulsed)	1.34E-02	1.00E-09	1.34E-11	5e-3 x 5e-3 mm	0.000025
SOT switching 2nm (pulsed)	1.64E-04	2.50E-10	4.1E-14	5e-3 x 5e-3 mm	0.000025
SOT switching 20nm (pulsed)	3.87E-01	2.50E-10	9.68E-11	5e-3 x 5e-3 mm	0.000025
RF source (0 dBm, sequential 200 channels)	1.00E-03	2.00E-06	2.00E-09	4 x 4 mm	16
RF source (noise source, parallel)	1.50E-01	1.00E-08	1.50E-09	20 x 20 mm	400

Supplementary Table 3. Power, time, energy and area of the various components required to operate projected nanomagnetic arrays.

required to switch. The coercive field of the nanomagnets also increases as thickness increases (we estimate up to 4 X based on micromagnetic simulations), as such we can expect between 10 – 40 X increase. This equates currents in the range of 25 – 100 mA and powers in the range of 16.8 – 269 mW range. Therefore, it may be preferable to use the Oersted field switching scheme for thicker nanomagnets.

Supplementary Table 3 shows the power, energy, time and area of the various components required to make a projected device. From this, we can calculate the power and energy of different device architectures. Supplementary Figure 24 shows two possible device architectures.

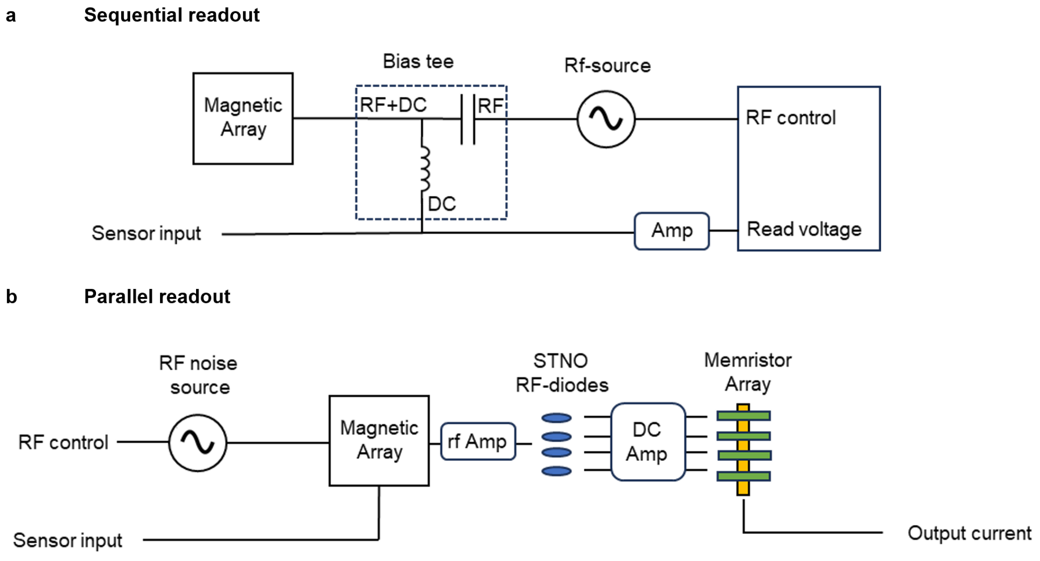

Supplementary Figure 24. Device schematic. a) Schematic of sequential spin-torque ferromagnetic resonance readout device. Sensory input is provided through a bias tee. Readout utilises spin-torque ferromagnetic resonance whereby a voltage is generated from spin-wave precession. b) Schematic of the parallel readout scheme where an RF noise source excites all modes in parallel. Signal is then passed to spin torque nano-oscillator (STNO) RF-diodes and then to a memristor array for weight multiplication.

We begin with a scheme which sequentially measures each frequency channel illustrated (Supplementary Figure 24 a)). In this scheme, a sensor input in the form of a current is applied to the device via a bias tee. Following this, a broadband RF source is swept and the DC voltage output at each frequency is recorded. The power energy and time when operating this scheme is presented below in Supplementary Table 4 for the two input schemes (ignoring DC amplification for now).

In this scheme, the time and energy for one update is dominated by the RF readout. Total power is dependent on the input scheme. This scheme requires temporary storage of each channel in a separate memory cache which would add to the power, time, and energy. A simple, but unoptimized, way of achieving this would be to connect the hardware to a low-power microcontroller such as an Arduino Uno operating at 100 mW.

Alternatively, we can consider the parallel readout scheme illustrated in Supplementary Figure 24 b). In this scheme, a sensory input is applied to the device to switch the state. An rf-noise source simultaneously excites all frequencies, and the

Input	Power (W)	Time (s)	Energy (J)
Oersted field	1.44E-02	2.80E-06	2.80E-09
SOT switching (2nm)	1.17E-03	2.80E-06	2.80E-09

Supplementary Table 4. Power, time, and energy for the sequential readout scheme for Oersted field and spin-orbit torque (SOT) switching.

resulting signal is sent to a series of spin-torque nano oscillator RF diodes tuned to different frequencies, serving as RF diodes. The spin-torque nano oscillators convert the RF signal into a set of DC voltages which are then passed, to a memristor array for weight multiplication. For input and readout, Supplementary Table 5 shows the powers, energies, and update times for the two different input schemes.

Input	Power (W)	Time (s)	Energy (J)
Oersted field	1.63E-01	1.50E-08	2.11E-09
SOT switching (2nm)	1.50E-01	1.43E-08	2.10E-09

Supplementary Table 5. Power, time, and energy for the parallel readout scheme for Oersted field and spin-orbit torque (SOT) switching.

Here, the time per update has reduced by two orders of magnitude compared to the previous scheme as the readout has been parallelised. On the contrary, the power has now increased by one order of magnitude and is dominated by the RF source. The energy per update in this scheme has reduced slightly.

We must also consider the power, energy, time and area required from any amplifiers in the circuit. In the sequential readout scheme (Supplementary Figure 6 a)) an amplifier is necessary to convert the low amplitude output voltage to a compatible read voltage for the microcontroller (we assume the sensory input is appropriately scaled to be compatible with input). The output voltage from the arrays at a power of 0 dBm is in the range of 1 – 10 µV which must be scaled to 0 – 5 V range. The required amplification to convert a 10 µV signal to 5 V is 5 / 10e-6 = 550,000. Low power instrumental amplifiers operating at $\sim$ 10 µW powers have been demonstrated with the necessary gains[24] and chip sizes around 4 mm^2. Time and energy for this amplification would have minimal impact on the previously calculated values.

For the parallel readout scheme (Supplementary Figure 24 b)), we require a series of DC amplifiers in between the spin torque nano oscillators and the memristor array. We can use the above DC amplifiers, requiring a total power of $N \times 10\,\mu$W, where N is the number of channels measured (2 mW for 200 channels). This process requires stable precession in the spin torque nano oscillator which takes 14 ns, giving an energy of 2.8e-11 J. As such, the time per update would double to $\sim$ 30 ns, whereas the energy would stay approximately the same.

In both the conventional hardware and nanomagnetic hardware, analogue to digital conversion is required. For conventional hardware, data gathered from a sensor will undergo conversion, with processing performed digitally. For nanomagnetic hardware, processing takes place in the analogue domain with readout being converted. For the sequential scheme, each frequency value will need to be converted whereas in the parallel scheme, only the readout value requires conversion. Therefore, the energy of the sequential scheme will increase as a result. Converters with high sample rate (1 GS/s) and operating powers or 11 mW are commonly available[25].

We can summarise the findings by comparing the four CMOS processors to the sequential and parallel schemes in Supplementary Figure 25.

We find that the time for a single update is slower than conventional hardware for small ESNs but more comparable for large ESNs. As nanomagnetic systems are further optimised to function as well as large ESNs, then the nanomagnetic schemes will become advantageous. Our projected devices are lower energy than conventional hardware (except GPUs) even for small ESN sizes. In terms of power, our schemes are $\sim$16– 2,100 X lower compared to Zynq FPGA hardware. The conventional hardware powers here are quoted as base powers, as opposed to the power required to update the ESN. The conventional hardware can be made to operate at lower powers, but at an additional time cost.

So far, we have considered single arrays. When creating a PNN using conventional hardware, the energy and time costs scale linearly with the number of echo-state networks, as adding an additional network requires the same number of FLOPs to input and update. The total power remains the same.

For the nanomagnetic arrays, adding additional reservoirs to the sequential scheme would require a series of relay switches to control where the input signal is sent at any given time (Supplementary Figure 26). This could, in principle, be achieved with transistors or MEMS components. The RF signals can also be sent to certain locations via rf-mems components. These components require 0.1 mW to operate and hence do not greatly affect the overall power consumption of the proposed devices.

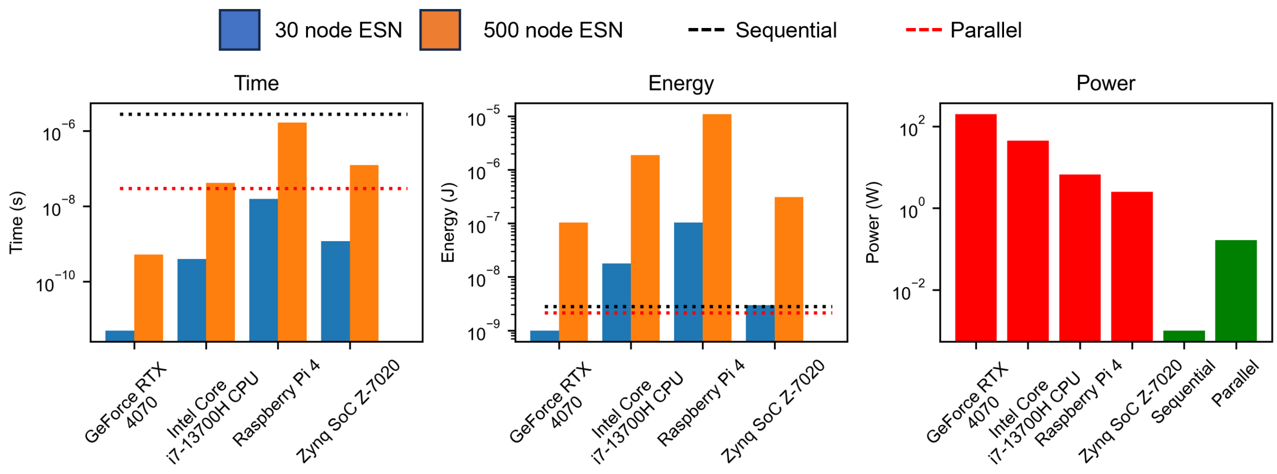

Supplementary Figure 25. Performance comparison. Comparison of the power, time and energy of updating an ESN vs our projected nanomagnetic devices

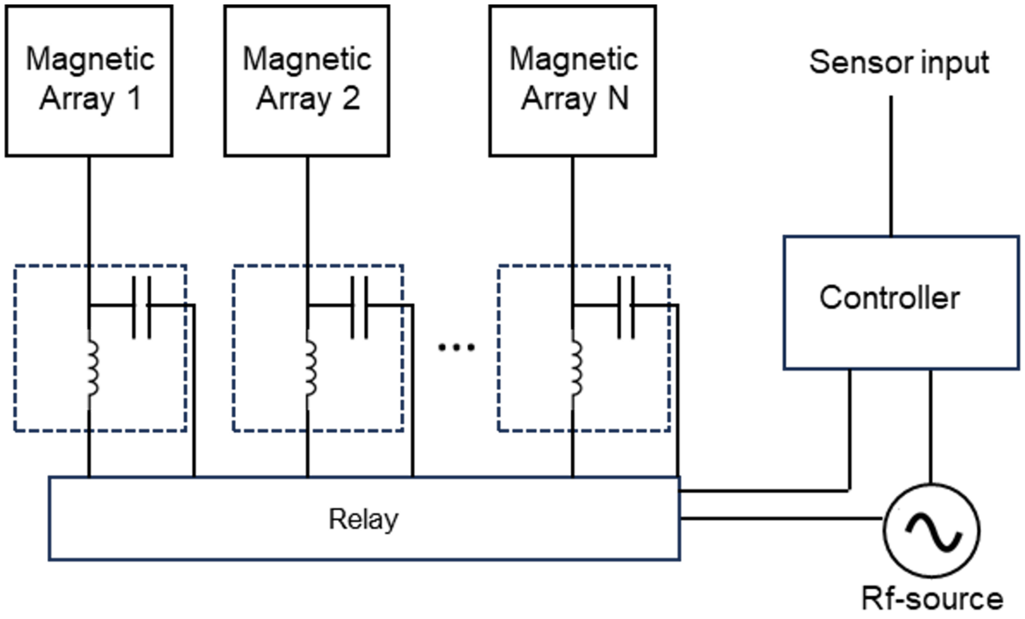

Supplementary Figure 26. PNN device schematic. Example schematic of a hardware PNN with sequential RF readout method.

In this case, the time and energy would by a factor of N larger, where N is the number of magnetic arrays. Here, the area of the device remains dominated rf-source. For the parallel readout scheme, each array would need to be sequentially measured to receive input from the previous array in the network. As such, we again find that the time and energy would increase by a factor of N for this system.

In summary, in both software and hardware options, the energy and time scales in the same manner with increasing PNN size, therefore the previous comparison of performance and energy with a single array is sufficient for comparison. Whilst the focus here is on nanomagnetic reservoirs, our PNN scheme can be used with other low-power / low-energy neuromorphic technologies. One example is low power memristor arrays which run at $\sim$22 µW[26]. If one were to interconnect these dynamic

memristors into PNNs, one can benefit from the computational advantages of the networked approach, at a fraction of the power consumption of our proposed devices.

Supplementary note 14 - Architecture details

Network	Data # on repo.	Figure
MS	0	1i, 2f,g, 3a-d
WM	7	1i, 2f,g, 3a-d, 4f
PW	14	1i, 2f,g, 3a-d
Parallel (MS + PW + WM)	0,7,14 (Pall)	1i, 2f,g, 3a-d
2 series (MS → WM)	0, 36 (S34)	1i, 2f,g, 3a-d
3 series (MS → WM → PW)	0, 36, 47 (S45)	1i, 2f,g, 3a-e
PW→WM	14, 17 (S15)	2f
PW→PW	14, 25 (S23)	2f
WM→MS	7, 29 (S27)	2f
MS→PW→WM	0, 18, 41 (S39)	2f

Supplementary Table 6. Table of architectures used in the main figures of the manuscript. The network architecture and sub-reservoirs are displayed in the 'Network' column. The data numbers and architecture names from the GitHub repository are displayed under 'Data # on repo.'. The figures which contain each network are displayed under 'Figure'.

Supplementary Table 6 displays the network, data labels and figures which contain this network. Data numbers and network names match those within the 'Data' folder of the GitHub repository.

Supplementary References

1. Skjærvø, S. H., Marrows, C. H., Stamps, R. L. & Heyderman, L. J. Advances in artificial spin ice. *Nat. Rev. Phys.* **2**, 13–28 (2020).
2. Gartside, J. C. *et al.* Reconfigurable training and reservoir computing in an artificial spin-vortex ice via spin-wave fingerprinting. *Nat. Nanotechnol.* **17**, 460–469 (2022).
3. Macêdo, R., Macauley, G., Nascimento, F. & Stamps, R. Apparent ferromagnetism in the pinwheel artificial spin ice. *Phys. Rev. B* **98**, 014437 (2018).
4. Li, Y. *et al.* Superferromagnetism and domain-wall topologies in artificial "pinwheel" spin ice. *ACS nano* **13**, 2213–2222 (2018).
5. Schultheiss, K. *et al.* Excitation of whispering gallery magnons in a magnetic vortex. *Phys. review letters* **122**, 097202 (2019).
6. Jaeger, H. Adaptive nonlinear system identification with echo state networks. *Adv. neural information processing systems* **15** (2002).
7. Dambre, J., Verstraeten, D., Schrauwen, B. & Massar, S. Information processing capacity of dynamical systems. *Sci. reports* **2**, 1–7 (2012).
8. Inubushi, M. & Yoshimura, K. Reservoir computing beyond memory-nonlinearity trade-off. *Sci. reports* **7**, 1–10 (2017).
9. Goldmann, M., Köster, F., Lüdge, K. & Yanchuk, S. Deep time-delay reservoir computing: Dynamics and memory capacity. *Chaos: An Interdiscip. J. Nonlinear Sci.* **30**, 093124 (2020).
10. Love, J. *et al.* Spatial analysis of physical reservoir computers. *Phys. Rev. Appl.* **20**, 044057 (2023).
11. Manneschi, L. *et al.* Exploiting multiple timescales in hierarchical echo state networks. *Front. Appl. Math. Stat.* **6**, 76 (2021).
12. D'Souza, R. M., di Bernardo, M. & Liu, Y.-Y. Controlling complex networks with complex nodes. *Nat. Rev. Phys.* 1–13 (2023).
13. Manneschi, L., Lin, A. C. & Vasilaki, E. Sparce: Improved learning of reservoir computing systems through sparse representations. *IEEE Transactions on Neural Networks Learn. Syst.* (2021).
14. Packard, N., Crutchfield, J., Farmer, D. & Shaw, R. Geometry from a time series. *Phys. Rev. Lett.* **45**, 712–716 (1980).

15. Takens, F. Detecting strange attractors in turbulence. *In D. A. Rand L.-S. Young (ed.). Dyn. Syst. Turbul. Lect. Notes Math. Springer-Verlag* **898**, 366–381 (1981).

16. Thorne, B., Juengling, T., Small, M., Correa, D. & Zaitouny, A. Reservoir time series analysis: Using the response of complex dynamical systems as a universal indicator of change. *Chaos: An Interdiscip. J. Nonlinear Sci.* **32** (2022).

17. Nvidia geforce rtx 4070 specs. https://www.techpowerup.com/gpu-specs/geforce-rtx-4070.c3924. Accessed: 2024-04-26.

18. Intel core i713700h processor 24m cache up to 5.00 ghz product specifications. https://ark.intel.com/content/www/us/en/ark/products/232128/intel-core-i7-13700h-processor-24m-cache-up-to-5-00-ghz.html. Accessed: 2024-04-26.

19. The gflops/w of the various machines in the vmw research group. https://web.eece.maine.edu/~vweaver/group/green_machines.html. Accessed: 2024-04-26.

20. Processing, B. D. S. GPU vs FPGA Performance Comparison. Tech. Rep., Berten Digitial Signal Processing (2016).

21. Ross, A. *et al.* Multilayer spintronic neural networks with radiofrequency connections. *Nat. Nanotechnol.* 1–8 (2023).

22. Kiermaier, J., Breitkreutz, S., Csaba, G., Schmitt-Landsiedel, D. & Becherer, M. Electrical input structures for nanomagnetic logic devices. *J. Appl. Phys.* **111** (2012).

23. Manchon, A. *et al.* Current-induced spin-orbit torques in ferromagnetic and antiferromagnetic systems. *Rev. Mod. Phys.* **91**, 035004 (2019).

24. Kim, J. & Ko, H. A dynamic instrumentation amplifier for low-power and low-noise biopotential acquisition. *Sensors* **16**, 354 (2016).

25. Zahrai, S. A. & Onabajo, M. Review of analog-to-digital conversion characteristics and design considerations for the creation of power-efficient hybrid data converters. *J. Low Power Electron. Appl.* **8**, 12 (2018).

26. Zhong, Y. *et al.* A memristor-based analogue reservoir computing system for real-time and power-efficient signal processing. *Nat. Electron.* **5**, 672–681 (2022).
